# Evaluating cellular roles and phenotypes associated with trehalose degradation genes in *Saccharomyces cerevisiae*

**DOI:** 10.1093/g3journal/jkae215

**Published:** 2024-09-09

**Authors:** Anqi Chen, Sara E Stadulis, Kayla deLeuze, Patrick A Gibney

**Affiliations:** Department of Food Science, Cornell University, Ithaca, NY 14853, USA; Science Center for Future Foods, Jiangnan University, Wuxi 214122, China; Department of Food Science, Cornell University, Ithaca, NY 14853, USA; Department of Food Science, Cornell University, Ithaca, NY 14853, USA; Department of Food Science, Cornell University, Ithaca, NY 14853, USA

**Keywords:** trehalose degradation, trehalase, *Saccharomyces cerevisiae*, genetic heterogeneity, *NTH1*, *NTH2*, *ATH1*

## Abstract

In the yeast *Saccharomyces cerevisiae*, 2 types of trehalase activities have been described. Neutral trehalases (Nth1 and Nth2) are considered to be the main proteins that catalyze intracellular trehalose mobilization. In addition to Nth1 and Nth2, studies have shown that acid trehalase Ath1 is required for extracellular trehalose degradation. Although both neutral and acid-type trehalases have been predominantly investigated in laboratory strains of *S. cerevisiae*, we sought to examine the phenotypic consequences of disrupting these genes in wild strains. In this study, we constructed mutants of the trehalose degradation pathway (*NTH1*, *NTH2*, and *ATH1*) in 5 diverse *S. cerevisiae* strains to examine whether published lab strain phenotypes are also exhibited by wild strains. For each mutant, we assessed a number of phenotypes for comparison to trehalose biosynthesis mutants, including trehalose production, glycogen production, cell size, acute thermotolerance, high-temperature growth, sporulation efficiency, and growth on a variety of carbon sources in rich and minimal medium. We found that all trehalase mutants including single deletion *nth1*Δ, *nth2*Δ, and *ath1*Δ, as well as double deletion *nth1nth2*Δ, accumulated higher intracellular trehalose levels compared to their isogenic wild-type cells. Also, *nth1*Δ and *nth1*Δ*nth2*Δ mutants exhibited mild thermal sensitivity, suggesting a potential minor role for trehalose mobilization when cells recover from stress. In addition, we evaluated phenotypes more directly relevant to trehalose degradation, including both extracellular and intracellular trehalose utilization. We discovered that intracellular trehalose hydrolysis is critical for typical spore germination progression, highlighting a role for trehalose in cell cycle regulation, likely as a storage carbohydrate providing glycolytic fuel. Additionally, our work provides further evidence suggesting Ath1 is indispensable for extracellular trehalose utilization as a carbon source, even in the presence of *AGT1*.

## Introduction

*Saccharomyces cerevisiae*, the budding yeast, is a model organism for investigating fundamental aspects of eukaryotic cell biology (Duina *et al.* 2014). Among *S. cerevisiae* research studies, many utilize a small number of strains adapted to laboratory growth and manipulation, which has proven useful for comparing results between studies in identical strains. One common strain lineage started with the S288C strain, which was isolated by Robert Mortimer, and its derivatives are among the most widely used laboratory strains of *S. cerevisiae* (Mortimer and Johnston 1986; Engel *et al.* 2014). This strain background has been useful for studying mutation effects and gene functions, it was the first eukaryotic organism to have its genome completely sequenced, and it has been the source of multiple strain collections such as overexpression and deletion mutant libraries (Mortimer and Johnston 1986; Goffeau *et al.* 1996; Giaever *et al.* 2002; Huh *et al.* 2003; Sopko *et al.* 2006). However, this reference strain, like any genetic background, only represents a subset of the many aspects of natural yeast biology, highlighting the risk of extrapolating gene–trait correlations observed in lab-domesticated lineages to species as a whole (Warringer *et al*. 2011; Su *et al*. 2021; Scott *et al*. 2023). Although laboratory strains such as the S288C derivatives remain important for studying fundamental elements of eukaryotic biology, investigating the phenotypic variation of *S. cerevisiae* wild isolates can also be useful to identify both the phenotypic variability within strains of the same species and the genetic basis underlying observed phenotypic variance. As an example, we recently evaluated phenotypic consequences of trehalose biosynthesis mutants in a number of wild yeast strains compared to a laboratory yeast strain, which yielded multiple instances of strain-to-strain variation that enable evaluation of the genetic basis of respective phenotypes (Chen *et al.* 2022).

**Fig. 1.**
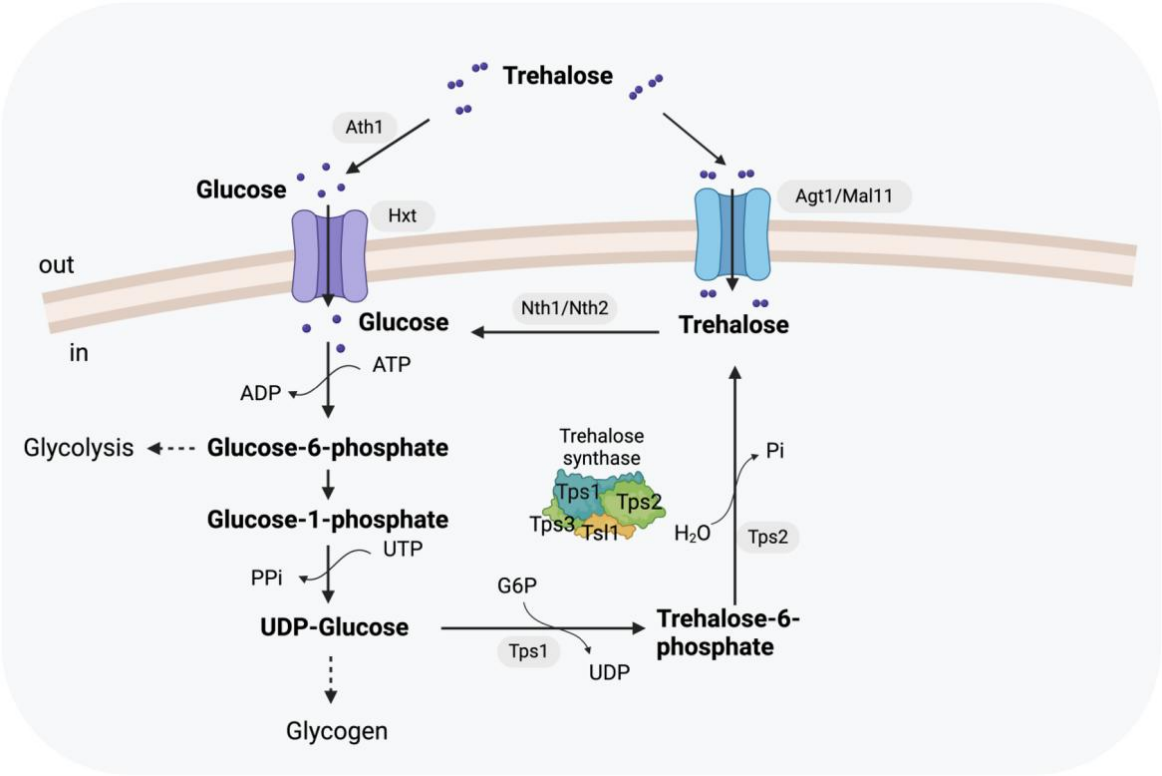
Schematic of trehalose metabolism in *S. cerevisiae*. Major metabolites (bold), enzymes/proteins (highlighted in gray), and other reactants/products are indicated. Additionally, branching pathways for glycolysis and glycogen synthesis are indicated. Tps3 and/or Tsl1 have proposed roles in supporting Tps1 and/or Tps2 function, though the mechanism is not clearly understood.

Trehalose is a nonreducing disaccharide in which 2 glucose molecules are connected with a α-1,1-glycosidic linkage. Trehalose metabolism is widely distributed in various organisms, including bacteria, fungi, plants, insects, and invertebrates (Elbein *et al.* 2003). In the yeast *S. cerevisiae*, trehalose may constitute as much as 15–20% of its dry weight when undergoing environmental stress (Van Dijck *et al.* 1995). While yeast cells actively growing on rich carbon sources contain very low levels of trehalose, as they enter the stationary phase when nutrients are exhausted or during growth on nonfermentable carbon sources, the amount of intracellular trehalose substantially increases (Yi *et al.* 2016). However, when nutrients are replenished, trehalose is rapidly converted to glucose (Nwaka and Holzer 1997). In *S. cerevisiae*, trehalose hydrolysis to glucose depends on 2 types of hydrolase enzymes: the neutral trehalases (Nth1 and Nth2) and acid trehalase (Ath1), named due to optimal pH for enzymatic activity (Fig. 1) (Jules *et al.* 2008). The trehalose degradation pathway has been examined in multiple laboratory strains, including S288C, CEN.PK, and SEY6210, among others, and this work has characterized the major genes and proteins involved in yeast trehalose degradation (Nwaka, Mechler, *et al.* 1995; Jules *et al.* 2004; Gibney *et al.* 2015). A wealth of information exists on the neutral trehalase enzymes Nth1 and Nth2, including their role in regulating intracellular trehalose levels within *S. cerevisiae* as well as characterization in other fungi (Thevelein 1984; Nwaka and Holzer 1997). Nth1 and Nth2 localize to the cytosol and have maximal activity at pH 6.8–7; Nth2 shares 77% identity at the amino acid level with Nth1 (Nwaka, Kopp, *et al.* 1995; Nwaka, Mechler, *et al.* 1995). Deletion of *NTH1* prevents intracellular trehalose hydrolysis and a loss of measurable trehalase activity (Nwaka and Holzer 1997). *NTH2* was found to be expressed at low levels in exponentially growing cells on glucose and at high levels in stationary phase after glucose exhaustion (Nwaka, Kopp, *et al.* 1995).

*ATH1* encodes an acid trehalase enzyme with an optimal activity at pH 4.5–5 (Destruelle *et al.* 1995). *Saccharomyces cerevisiae*Ath1 includes an N-terminal signal peptide and transmembrane domain (Parrou *et al.* 2005). Deletion of *ATH1* prevents cells from utilizing extracellular trehalose as a carbon source (Nwaka *et al.* 1996; Gibney *et al.* 2015). Acid trehalase activity has been detected in glucose-grown stationary phase cells and on respiratory substrates, suggesting its activity may be subject to glucose repression, though its association with the general stress response is unlikely because no stress response element (STRE) sequences have been observed in the *ATH1* promoter (San Miguel and Argüelles 1994). Divergent observations regarding Ath1 localization have led to multiple models for extracellular trehalose consumption: one model proposes that extracellular Ath1 hydrolyzes trehalose into glucose before glucose import, while another model proposes that trehalose is first imported into the cell and then the vacuole where it is hydrolyzed to glucose by vacuolar-localized Ath1. Despite open questions related to the localization of trehalose catabolism by Ath1, there is a clear consensus that Ath1 is required for utilization of extracellular trehalose (Nwaka *et al.* 1996; Nwaka and Holzer 1997; Huang *et al.* 2007; He *et al.* 2009).

The main goal of this work was to evaluate phenotypic diversity of trehalose degradation mutants in wild strains compared to a lab strain and then use this information to better understand the physiological roles associated with trehalose degradation enzymes. To accomplish this goal, we separately deleted each of the 3 genes encoding proteins for trehalose mobilization (*NTH1*, *NTH2*, and *ATH1*), including a double gene deletion mutant of the neutral trehalase genes and a triple gene deletion mutant of all 3 trehalase genes. We constructed these deletion mutants in 2 commercial wine strains (Simi White and CSM), 1 vineyard isolate [Bb32(3)], 1 oak tree isolate (YPS1000), and an S288C derivative. With regard to phenotypic characterization, we employed 2 strategies. First, we evaluated a series of phenotypes that were previously evaluated with a panel of trehalose biosynthesis mutants made in the same strains; these phenotypes included stationary phase intracellular trehalose and glycogen concentrations, carbon source utilization, high-temperature survival and growth, and sporulation efficiency (Chen *et al.* 2022). This phenotypic characterization allows for comparing effects of failing to produce intracellular trehalose in biosynthesis mutants with the effects of failing to degrade intracellular trehalose in trehalose degradation mutants. Such comparisons can be helpful to identify phenotypes that are directly related to trehalose levels. Second, we evaluated a series of phenotypes that are more directly relevant to trehalose degradation mutants, including extracellular trehalose utilization and intracellular trehalose utilization during both lag phase and spore germination.

Phenotypic characterization of these trehalose degradation mutants has led to a number of insights, including confirmation of lab strain phenotypes in wild strains, identification of novel phenotypes, and conceptual models for the roles of trehalose and trehalases in cellular physiology. For example, spores from *nth1*Δ*nth2*Δ strains exhibited a germination defect, indicating the importance of intracellular trehalose utilization to fuel exit from the arrested cell cycle associated with the spore state. Further, mutant strains lacking *NTH1* exhibited mildly compromised thermotolerance, suggesting the importance of intracellular trehalose degradation when cells are recovering from thermal stress. As the localization and role of Ath1 in intracellular trehalose degradation have been controversial, we also present data exploring the function of Ath1. We confirmed that Ath1 is indispensable to utilizing extracellular trehalose, even in a strain that encodes *AGT1*, a disaccharide transporter. These results suggest that Agt1 may not be a typical route for natural trehalose import. In addition, slightly increased intracellular trehalose was found in many *ath1*Δ mutants, even with cytoplasmic trehalases present, illustrating the potential role of Ath1 in vacuolar degradation of trehalose. However, whether vacuolar trehalose or its metabolism has any physiological consequences remains unclear. We demonstrate that Ath1 is not involved in cytosolic trehalose degradation, as accumulated intracellular trehalose in *nth1*Δ *nth2*Δ mutants is not degraded in strains with wild-type or overexpressed *ATH1*. Altogether, these results compare trehalose degradation mutant phenotypes across diverse genetic backgrounds, highlight an important role for trehalose and trehalose degradation in spore germination, and clarify the role of Ath1 in extracellular trehalose utilization. This work provides a well-characterized panel of trehalose degradation mutants and expands the knowledge on how systems regulating intracellular and extracellular trehalose mobilization are connected.

## Materials and methods

### Yeast growth media

Yeast cell growth and genetic manipulations were performed using standard approaches (Guthrie and Fink 1991). All media used were either minimal (YNB: 0.67% w/v yeast nitrogen base without amino acids plus 2% w/v indicated carbon sources) or rich (YP: 2% w/v bactopeptone, 1% w/v yeast extract, 2% w/v indicated carbon sources). Exceptions are YPGE and SGE media, rich and minimal formulations, respectively, containing both 3% w/v glycerol and 2% w/v ethanol as respiratory carbon sources. YPD indicates rich media containing 2% glucose (dextrose). Solid media formulations included 2% w/v agar and were poured into standard round 10 cm plates or rectangular plates (Petri 1887).

### Yeast growth evaluation

Cell concentration estimation was evaluated by measuring transmittance of 600 nm light (OD_600_) through a cell suspension using a Gensys 6 UV-Vis spectrophotometer (Thermo Fisher) or using a Synergy H1 Hybrid reader (BioTek). For plate reader growth, 200 µL cultures were prepared in a clear, flat-bottom, Corning Costar 96-well plate; plates were sealed with a Breathe-Easy gas-permeable membrane from Research Products International Corporation. The 96-well plate was incubated at 30 °C with a double orbital shaking at a speed of 559 cpm. Measurements of cell density and cell size were performed using a Coulter Z2 Particle Count and Size Analyzer (Beckman Coulter) with a 100-µm aperture. Cell size measurements were converted from a spherical diameter to volume for reporting. For comparative growth assays, cell dilutions were spotted onto relevant solid growth media. Cell spotting was performed by dilution of a stationary phase culture to an initial OD_600_ of 1.0, followed by 10-fold serial dilutions. All dilutions were then spotted onto solid media using a replica plater for 96-well plate, either the 8 × 6 or 12 × 8 array as needed (Sigma-Aldrich). Plates were incubated at indicated temperatures for times noted in respective figures and captions. For growth in minimal media containing trehalose or maltose, cells were first grown to stationary phase in YNB + 2% glucose before being diluted to OD_600_ of 0.05 in minimal trehalose/maltose media. OD_600_ measurements were then taken every day for a total of 12 days. At least 3 independent biological replicates were performed on different days for spotting assays shown in figures, and a representative image is shown.

### Yeast strain construction

The strains used in this study are listed in Supplementary Table 1. Gene deletions were constructed by transforming PCR products amplified from plasmids containing different deletion cassettes: pFA6a-kanMX for kanMX, pAG32 for hphMX, and pAC372 for natAC (Supplementary Table 2). Primers were designed with 40 flanking base pairs identical to the upstream and downstream of genes to be deleted by homologous recombination. All gene deletions in the S288C background (*GAL*^+^, *HAP1*-repaired, prototrophic derivatives) were made by transformation into a diploid to yield a heterozygous gene deletion, which was confirmed by PCR then dissected to get MAT**a** and MATα segregants before mating together obtain a homozygous diploid. Similarly, mutant strains constructed from non-S288C strains (Simi White, YPS1000, CSM, and Bb32) were made in the same way, although homozygous diploids were obtained directly after tetrad dissection (the wild strains are homothallic; thus, after spores germinate, cells can switch mating types and mate to produce colonies that are essentially all diploid cells). All gene deletions were confirmed by PCR (Supplementary Fig. 1). All combinatorial gene deletion/insertion strains were made by mating, sporulating, and tetrad dissection, followed by selective plating. Sporulation was performed by growing cells to log phase in rich media, collecting cells by centrifugation, washing once in 1% w/v potassium acetate, and then resuspending in 1% w/v potassium acetate. Cells were then incubated at room temperature on a roller wheel for at least 6 days before tetrad dissection.

### Plasmid construction

The plasmids used in this study are listed in Supplementary Table 2. Plasmids were built using pRS series shuttle vector backbones containing the *THD3* promoter and *CYC1* 3′UTR (Sikorski and Hieter 1989; Mumberg *et al.* 1995). All inserts were amplified using primers from Integrated DNA Technologies containing 5′end SpeI and 3′end XhoI sites to use for restriction enzyme-based confirmation of inserts during cloning. *ATH1* expression plasmids were cloned using Gibson assembly: plasmids p416GPD and p426GPD were linearized using SpeI and XhoI, then incubated with PCR inserts containing *ATH1* amplified from DBY12007 genomic DNA along with the Gibson Assembly mixture at 50 °C for 1 h before transforming into TOP10 *Escherichia coli* (Gibson *et al.* 2009). After transformation, individual colonies were screened for correct insertion using restriction digest. All gene insertion and allele-specific mutations were confirmed by Sanger sequencing at the Cornell Institute of Biotechnology sequencing core facility.

### Assessment of thermotolerance

To assess thermotolerance, minimal media cultures were inoculated with a single colony and grown overnight to stationary phase. To standardize the growth regime leading to a stationary phase culture, cells were then diluted into fresh minimal medium to an OD_600_ = 0.05 and grown another 24 h to stationary phase. Two aliquots of 0.8 mL cell culture were then removed into microcentrifuge tubes. For the heat shock, one of the aliquots was incubated in a 42 °C thermomixer for 2 h. Both pre- and postheat shocked cell dilutions were plated on rich media (YPD) and incubated at 30 °C for 2–3 days to measure viability by counting colony forming units. At least 3 independent biological replicates were performed for each thermotolerance assay.

### Measurement of sporulation efficiency

Sporulation was performed by growing cells to log phase in rich media (except plasmid-containing cells, which were grown in minimal media for plasmid maintenance), collecting cells by centrifugation, washing twice in 1% w/v potassium acetate, and then resuspending in 1% w/v potassium acetate. Cells were then incubated at room temperature on a roller wheel for at least 6 days. Sporulated cultures were evaluated by counting tetrads, or asci containing 4 spores; 2-spore- and 3-spore-containing asci, dyads or triads, respectively, were not observed in any significant fraction. Sporulation efficiency was calculated as the proportion of observed tetrads over the total number of observed cells, with a minimum of 300 total cells counted. At least 3 independent biological replicates were performed for each sporulation efficiency assay.

### Measurement of trehalose and glycogen

Trehalose and glycogen levels were measured essentially as described (Parrou and François 1997). Briefly, 10 OD_600_ units of stationary cells were harvested, washed in cold water, and resuspended in 250 µL of 0.25 M sodium carbonate. Cell mixtures were stored at −80 °C until the assay was performed. To begin the assay, cells were boiled at 95 °C for 4 h with occasional agitation—this step extracts the trehalose and glycogen, as both are highly stable and not heat-degraded (any residual glucose, however, is degraded under these alkaline, high-temperature conditions). Next, 150 µL of acetic acid was added to the sample, followed by 600 µL of 0.2 M sodium acetate. After mixing, two 350 µL aliquots were removed to fresh tubes, and either 5 µL of 70 U/mL trehalase (Megazyme) or 70 U/mL amyloglucosidase (Sigma-Aldrich) was added. These reactions were incubated overnight at 37 or 57 °C, respectively, in a thermomixer set at 550 rpm (Eppendorf). Next, the sample was clarified by centrifugation at maximum speed for 3 min, and 200 µL of each sample was used to measure the amount of glucose liberated from trehalose or glycogen using the Glucose (GO) Assay Kit (Sigma-Aldrich).

### Evaluation of germination

Germination was initially evaluated from sporulated cultures after being incubated in sporulation medium (1% w/v potassium acetate) at room temperature for 6 days. Six tetrads from each culture were dissected on YPD plates (24 total spores per culture), which were incubated at 30 °C for 8 h before the number of resulting cells from each germinated spore was counted by visual observation using the tetrad dissection microscope. To evaluate the time required for bud emergence from spores between strains and their respective trehalose mutants, 2 tetrads (8 spores) from the sporulated cultures were dissected on YPD plates and incubated at 30 °C. Through visual inspection each hour, we evaluated the number of hours required for bud emergence. Finally, time required for bud emergence of S288C-derived strains containing *ATH1* expression plasmids were similarly investigated to evaluate potential suppression activity of overexpressed *ATH1* on *nth1*Δ *nth2*Δ delayed bud emergence. Plasmid-containing strains were sporulated, and for each, 2 tetrads were dissected from 3 independent cultures, resulting in 24 spores per tested strain. Bud emergence time was evaluated as described above. A number of data points were not included in the resulting figure as some spores either failed to germinate within the 10-h assay window or failed to germinate after 3 days growth at 30 °C (0/24 spores removed from *ura3*Δ*0* + p416GPD, 1/24 removed from *ura3*Δ*0* + p416GPD-*ATH1*, 9/24 spores removed from *nth1*Δ *nth2*Δ *ura3*Δ*0* + p416GPD; 9/24 spores were removed from *nth1*Δ *nth2*Δ *ura3*Δ*0* + p416GPD-*ATH1*).

### Statistical analysis

All experiments were conducted using at least 3 independent biological replicates. Mutant phenotypes were evaluated for statistical significance compared to their isogenic wild-type strains using a 2-tailed paired *t*-test. Asterisks (*) in figures indicate the mutant phenotype showed a difference (*P* < 0.05) compared to its isogenic wild type (*P*-values were not corrected for multiple hypothesis testing). Separately, to evaluate statistically significant phenotypic differences between the wild strains examined, or also between different strains with identical gene deletions, 1-way ANOVA with post hoc Tukey honest significant difference (HSD) tests were performed (Supplementary Tables 3–7). Corresponding to the F-statistic of 1-way ANOVA, indicated *P*-values lower than 0.05 suggest that one or more evaluated treatments were significantly different.

## Results and discussion

### Trehalose mutant phenotypes under consideration

Trehalose is a widely distributed carbohydrate in nature that is synthesized in *S. cerevisiae* in response to nutrient limitation and during certain environmental stress (Lillie and Pringle 1980; Crowe *et al.* 1984; Thevelein 1984; Tapia *et al.* 2015). To examine the phenotypic diversity of mutants in the trehalose mobilization pathway, we constructed single deletion mutants (*nth1*Δ, *nth2*Δ, and *ath1*Δ), 1 double deletion mutant (*nth1*Δ*nth2*Δ), and 1 triple deletion mutant (*nth1*Δ*nth2*Δ*ath1*Δ) in 5 different strains of *S. cerevisiae* (Supplementary Table 1). These strains included 2 commercial wine strains (Simi White and CSM), 1 vineyard isolate [Bb32(3)], 1 oak tree isolate (YPS1000), and an S288C derivative for comparison. We initially focused on a core set of relevant phenotypes that could be easily compared to phenotypes of trehalose biosynthesis mutants in the same strain backgrounds (Chen *et al.* 2022). By evaluating these phenotypes, we can compare the consequences of failing to hydrolyze trehalose (excess intracellular trehalose) to the consequences of failing to produce trehalose (absence of intracellular trehalose). These comparisons can then be used to evaluate hypotheses about the role of intracellular trehalose on different cellular processes. We first quantified both intracellular trehalose and glycogen levels in single and double deletion trehalase mutants. Further, as stress tolerance has been correlated to intracellular trehalose concentration, we assessed thermotolerance (42 °C for 2 h) and ability to grow at an elevated temperature (growth at 37 °C). We also examined sporulation efficiency, cell size, and growth on multiple carbon sources in both rich and minimal media as trehalose biosynthesis mutants exhibit phenotypes in these conditions (Neves *et al.* 1995; de Silva-Udawatta and Cannon 2001; Gancedo and Flores 2004; Walther *et al.* 2013). We additionally evaluated trehalase roles in hydrolysis of both extracellular and intracellular trehalose, as these phenotypes are particularly relevant to the function of the Nth1, Nth2, and Ath1 enzymes.

### Construction and phenotypic characterization of nth1Δ, nth2Δ, and nth1Δnth2Δ

**Fig. 2.**
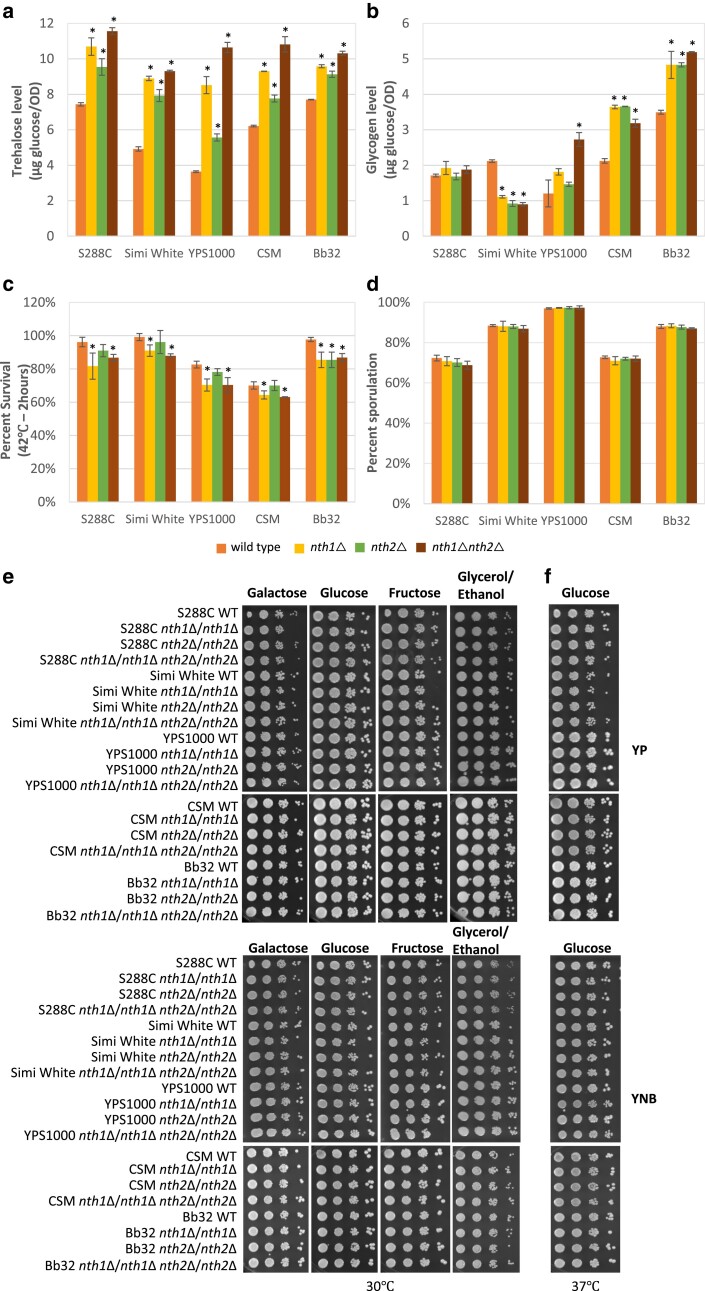
nth1Δ, nth2Δ, nth1Δ nth2Δ mutant phenotypes. a) Intracellular trehalose levels. b) Intracellular glycogen levels. c) Thermotolerance (heat shocked at 42 °C for 2 h). d) Sporulation efficiency. e and f) Growth at 30 and 37 °C on minimal (bottom panels) and rich (top panels) media. Indicated strains were grown overnight in YNB + 2% glucose liquid before 10-fold serial dilutions were prepared and spotted onto the indicated media. The initial dilution had an OD_600_ of 1.0. Plates were incubated at 30 °C for 2 days on rich media and 3 days for minimal media. Three biological replicates were performed for all tested phenotypes. Asterisks represent statistically significant differences (*P* < 0.05) between the mutants and their isogenic wild-type strains.

In *S. cerevisiae*, degradation of cytoplasmic trehalose is mainly catalyzed by neutral trehalase enzymes, encoded by *NTH1* and *NTH2* (Fig. 1) (Nwaka, Mechler, *et al.* 1995). Transcriptional activation of *NTH1* depends on the general stress response mediated by the Msn2/Msn4 transcription factors through the STRE elements present in its promoter (Thevelein 1984; Zähringer *et al.* 2000; Panni *et al.* 2008). The Nth enzymes are also subject to activation through posttranslational modification (Alblova *et al.* 2019). In the present study, all *nth1*Δ and *nth2*Δ mutants exhibited increased trehalose levels compared to their isogenic wild-type strains (Fig. 2a). All 5 *nth2*Δ mutants accumulated trehalose to a lower extent than *nth1*Δ mutants, though still higher than their isogenic wild-type strains, highlighting that Nth2 plays a minor but significant role in intracellular trehalose degradation (Fig. 2a). All *nth1*Δ*nth2*Δ double deletion mutants exhibited additive effects from deleting both neutral trehalases as they accumulated the highest level of intracellular trehalose (Fig. 2a). This result agrees with multiple previous studies demonstrating that the absence of Nth1 results in yeast cells unable to breakdown intracellular trehalose (Thevelein 1984; Nwaka, Kopp, *et al.* 1995; Gibney *et al.* 2015). Also, increased trehalose levels in *nth2*Δ mutants align well with the previous finding that overexpression of *NTH2* in an *nth1* mutant resulted in an approximately 10-fold increase of trehalose-hydrolyzing activity, suggesting Nth2 is a functional trehalase (Jules *et al.* 2008). Previous work using S288C-derived lab strains demonstrated a negative correlation between intracellular trehalose and glycogen levels, suggesting that when trehalose biosynthesis is disrupted, UDP-glucose gets shunted to glycogen production (François *et al.* 1991; de Silva-Udawatta and Cannon 2001). However, here we observed that intracellular glycogen levels were less consistent: mutants in different genetic backgrounds exhibited less, equivalent, or more glycogen than their isogenic wild-type strains, suggesting glycogen levels are either unrelated to flux through trehalose metabolism or the relationship is more complex than overflow metabolism (Fig. 2b). It is not clear why trehalase degradation mutants resulted in variation in the glycogen levels, but this further suggests that intracellular trehalose and glycogen levels are not necessarily anticorrelated, as also observed in trehalose biosynthesis mutants (Chen *et al.* 2022). We did not observe any significant cell size variations with any of these mutants (Supplementary Fig. 2a).

Trehalose has been proposed to be a protective agent against a variety of abiotic stresses (Hottiger *et al.* 1987; Hounsa *et al.* 1998; Singer and Lindquist 1998a; Elbein *et al.* 2003). Multiple studies revealed the ability of trehalose to stabilize proteins in vitro by retaining their native conformation at elevated temperatures and suppressing aggregation of denatured proteins (Singer and Lindquist 1998a, 1998b). However, recent work using the Agt1 overexpression system demonstrated that increased intracellular trehalose did not correlate with increased heat resistance (Gibney *et al.* 2015). Therefore, to further investigate the role of trehalose in heat stress protection in vivo, we tested thermotolerance in *nth1*Δ, *nth2*Δ, and *nth1*Δ*nth2*Δ mutants by exposing the cells to 42 °C for 2 h. We found that none of the tested mutants had increased thermotolerance, despite higher intracellular trehalose levels. In contrast, all *nth1*Δ and *nth1*Δ *nth2*Δ mutants in all 5 strains exhibited mildly compromised thermotolerance when exposed to 42 °C for 2 h, ranging from 85.0 to 91.9% survival compared to their isogenic wild type (Fig. 2c). Four out of 5 *nth2*Δ mutants exhibited the same level of heat resistance as their isogenic wild types, except the Bb32 *nth2*Δ that had a survival rate similar to *nth1*Δ. Comparing results between Fig. 2a and c suggests that trehalose accumulation and increased thermotolerance do not correlate, further indicating that intracellular trehalose may not have a significant role in heat protection in vivo. In addition, mild thermosensitivity associated with these mutants suggests that trehalose degradation activity may play a minor role in cellular protection against heat, likely associated with recovery. Our data align with previous work demonstrating that *nth1*Δ*nth2*Δ cells were found to be more thermosensitive than wild-type cells when exposed to 48 °C for 60 min and 50 °C for 20 min (Nwaka, Mechler, *et al.* 1995; Gibney *et al.* 2015). A possible explanation for this phenotype is based on the observation that trehalose can interfere with protein folding in vitro, which is also used to explain the rapid hydrolysis of trehalose in wild-type cells following heat shock as a mechanism to allow cells to more rapidly refold proteins and recover from heat shock (Singer and Lindquist 1998a). In addition to examining acute thermotolerance, we evaluated the ability of these mutants to grow at an elevated temperature. None of the deletion mutants exhibited any growth defect when incubated at 37 °C (Fig. 2e and f; Supplementary Fig. 3). In addition to evaluating heat stress response, we observed that sporulation efficiency was unaffected in *nth1*Δ, *nth2*Δ, and *nth1*Δ*nth2*Δ mutants in any of the 5 tested genetic backgrounds (Fig. 2d).

We also examined whether the absence of neutral trehalase enzymes affects utilization of different carbon sources, which is a common phenotype associated with deletion of trehalose biosynthesis genes but found no noticeable growth defects with trehalase mutants (Fig. 2e). Trehalose biosynthesis mutants *tps1*Δ and *tps2*Δ exhibit defective growth on different carbon sources (highly fermentative carbon source growth defects for *tps1*Δ and respiratory carbon source growth defects for *tps2*Δ), and the severity of the growth defect is highly variable between strains (Navon *et al.* 1979; González *et al.* 1992; Stucka and Blázquez 1993; Walther *et al.* 2013; Gibney *et al.* 2015; Chen *et al.* 2022). The corresponding lack of carbon source utilization defects for the mutants tested here suggests that intracellular trehalose concentration is not relevant for carbon source utilization signaling. The observed phenotypes in trehalose biosynthesis mutants are likely more related to other hypotheses, including independent signaling roles for either Tps1 or trehalose-6-phosphate, among others (Chen *et al.* 2022). The absence of growth defects observed here is in contrast to a report using laboratory strain SEY6210, where *nth1*Δ grew poorly on rich media containing glycerol compared to its isogenic wild type (Nwaka, Mechler, *et al.* 1995). Notably, previous work has highlighted that multiple laboratory strains (CEN.PK, W303, and S288C) of *S. cerevisiae* are unable to use glycerol as a sole carbon source in a minimal medium, suggesting growth on rich media containing glycerol may involve utilization of additional carbon sources other than glycerol (Swinnen *et al.* 2013).

### Construction and phenotypic characterization of ath1Δ

**Fig. 3.**
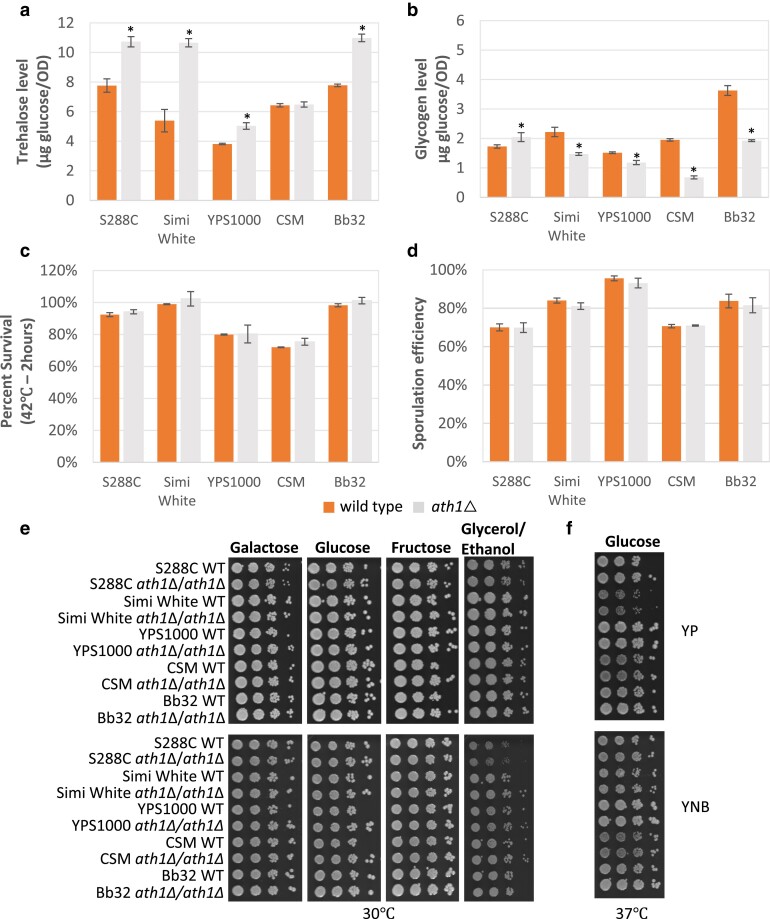
*ath1* Δ mutant phenotypes. a) Intracellular trehalose levels. b) Intracellular glycogen levels. c) Thermotolerance (heat shocked at 42 °C for 2 h). d) Sporulation efficiency. e and f) Growth at 30 and 37 °C on minimal and rich media. Indicated strains were grown overnight in YNB + 2% glucose liquid before 10-fold serial dilutions were prepared and spotted onto the indicated media. The initial dilution had an OD_600_ of 1.0. Plates were incubated at 30 °C for 2 days on rich media and 3 days for minimal media. Three biological replicates were used for all tested phenotypes. Asterisks represent statistically significant differences (*P* < 0.05) between the mutants and their isogenic wild-type strains.

*ATH1* encodes the acid trehalase enzyme with optimal activity at an acidic pH range (4.5–5) (Destruelle *et al.* 1995). We constructed *ath1*Δ mutants in all 5 genetic backgrounds and performed phenotype tests identical to those for the neutral trehalase mutants. Stationary phase intracellular trehalose levels in 4 of the 5 tested *ath1*Δ strains were significantly higher than their isogenic wild-type cells, ranging from 132 to 198%; CSM *ath1*Δ alone maintained wild-type levels of trehalose (Fig. 3a). Similarly, a previous study reported that *ath1*Δ in the S288C genetic background maintained higher intracellular trehalose accumulation than wild-type cells under both a saline-stress condition and in the postdiauxic phase during growth in 2% glucose; it was suggested that acid trehalase activities might be involved in intracellular trehalose metabolism under these stress conditions (Garre *et al.* 2009). Therefore, it is possible that Ath1 possesses dual roles in trehalose mobilization, both intracellular and extracellular, although if accurate, the functional role of intracellular trehalose degradation by Ath1 remains unclear. Glycogen levels of *ath1*Δ were slightly higher in the S288C strain, but lower in the 4 wild strains (Fig. 3b). We did not observe any significant cell size variations with any of the *ath1*Δ mutants (Supplementary Fig. 2b).

Deleting *ATH1* did not significantly compromise thermotolerance of any tested strain (Fig. 3c). Several previous studies investigated the effect of Ath1 absence on stress resistance, though some of them were difficult to interpret because they were affected by a confounding difference in an auxotrophic nutrient requirement associated with a *ura3*Δ mutation between mutant and reference strains (Nwaka, Mechler, *et al.* 1995; Kim *et al.* 1996; Chopra *et al.* 1999). Nwaka, Mechler, *et al.* (1995) detected no significant Ath1 activity from cells recovered from thermal stress, suggesting that unlike *NTH1*, the *ATH1* gene is not involved in the recovery of cells after heat shock. Notably *ATH1* transcription is not activated by stress likely due to lack of a STRE in its promoter, which mediates general stress-induced transcriptional activity by binding the Msn2/4 transcription factor (Destruelle *et al.* 1995; Nwaka *et al.* 1996). As with thermotolerance, none of the *ath1*Δ strains exhibited a growth defect when incubated at high temperature, 37 °C (Fig. 3f; Supplementary Fig. 4). Sporulation efficiency and carbon source utilization were similarly unaffected by the absence of Ath1 in any of the 5 tested genetic backgrounds (Fig. 3d and e).

### Comparing trehalose degradation phenotypes to trehalose biosynthesis phenotypes

It is noteworthy that many of the phenotypes evident in trehalose biosynthesis mutants are not evident in trehalose degradation mutants (Chen *et al.* 2022). Trehalose biosynthesis mutants evaluated with a similar panel of phenotypic tests exhibited heat survival defects, temperature-sensitive growth defects, sporulation defects, and carbon source utilization defects. Other than a mild defect in heat survival associated with *nth1*Δ, trehalose degradation mutants did not exhibit variability compared to wild-type cells for these phenotypes. Comparison of these phenotypes suggests that lack of trehalose itself is not responsible for the phenotypes described above associated with biosynthesis mutants. For example, accumulation of intracellular trehalose either by disruption of *NTH* genes or via import using constitutively expressed *AGT1* is sufficient to induce both desiccation tolerance and freeze–thaw tolerance (Tapia *et al.* 2015; Chen and Gibney 2022). Together, these results suggest that trehalose itself acts as a molecular chaperone under desiccation or freeze–thaw conditions, both conditions with low water activity that may align with the notion that trehalose can act as a molecular chaperone by replacing the hydrogen-bonding activity associated with water molecules (Olsson *et al.* 2016; Shao *et al.* 2019). In contrast, accumulation of trehalose does not appear to enhance heat survival, suggesting that trehalose itself may not act as a molecular chaperone for heat shocked cells in conditions with high intracellular water activity (Gibney *et al.* 2015). The absence of phenotypes observed for trehalose degradation mutants compared to biosynthesis mutants, along with repeated observation in multiple strains, provides a useful comparison to evaluate direct effects of trehalose. Multiple studies have sought to further distinguish the trehalose-independent roles of trehalose metabolism in carbon source utilization, heat survival, and sporulation, though detailed molecular mechanisms that incorporate known phenotypic data remain elusive (Gibney *et al.* 2015; Chen *et al.* 2022). Another contrast to the phenotypic characterization of trehalose biosynthesis mutants is strain-to-strain phenotypic variability. For most of the biosynthesis mutants, at least one strain failed to exhibit the phenotype associated with the other strains. This was not the case with trehalose degradation mutants, which exhibited more uniformity among phenotypes tested here. One notable exception, similar to trehalose biosynthesis mutants, was the variability in stationary phase intracellular glycogen concentrations between strains. It is possible that these variations are related to variations in stationary phase between strains, such as differential timing for stationary phase initiation or differential expression levels of glycogen metabolism enzymes.

### Extracellular trehalose utilization by Ath1

Beyond phenotypes useful for comparison with trehalose biosynthesis mutants, we sought to evaluate the consequences of trehalase gene deletion on a number of phenotypes directly relevant to conditions of enzymatic trehalose hydrolysis, including intracellular and extracellular trehalose utilization. Yeast growth using extracellular trehalose is relatively slow in a number of laboratory strain backgrounds (CEN.PK, S288C, and JF657). The assimilation of this disaccharide is purely oxidative, and in an S288C derivative background, it yields a doubling time of approximately 17 h (Nwaka *et al.* 1996; Jules *et al.* 2004; Gibney *et al.* 2015). Conflicting evidence regarding Ath1 localization and activity has resulted in multiple conceptual models for extracellular trehalose degradation. To investigate the cellular localization of Ath1, Huang *et al.* (2007) expressed a C-terminal, GFP-tagged Ath1 in cells grown in minimal glucose media to log phase, then used trafficking mutants to illustrate Ath1 reaches the vacuole via the multivesicular body pathway. They suggest a conceptual model wherein vacuolar Ath1 consumes extracellular trehalose in the vacuole. However, the publication also reports only roughly 50% of the acid trehalase activity is vacuolar, but did not specify localization of remaining activity (Huang *et al.* 2007). Furthermore, no evidence was presented regarding how extracellular trehalose is transported both into the cell and then into the vacuole. In contrast, He *et al.* (2009) used mCherry fused to Ath1 to demonstrate both vacuolar and plasma membrane localization. By disrupting delivery of Ath1 to the vacuole, this study demonstrated no effect on either Ath1 enzymatic activity or growth rate of cells on extracellular trehalose, indicating that vacuolar-localized Ath1 is not important for extracellular trehalose utilization (He *et al.* 2009). He *et al.* (2009) also demonstrated that fusing the signal peptide of invertase to an N-terminally truncated Ath1 missing its native signal sequence was sufficient to allow *ath1*Δ growth on trehalose, suggesting that functional localization of Ath1 at the cell periphery is required for extracellular trehalose mobilization. Additionally, fusion of the Ath1 signal sequence and transmembrane domain to an invertase allele lacking its own signal sequence and transmembrane domain is required to recover invertase activity and growth on sucrose in a *suc2*Δ mutant (He *et al.* 2009). This study supports the conceptual model that extracellular trehalose is hydrolyzed to glucose outside of the cell by extracellular Ath1. The 2 models are not mutually exclusive, as both mechanisms could be applicable. It is also possible that plasma membrane localization represents functional expression of Ath1, while vacuolar-localized Ath1 is merely a consequence of membrane recycling and is not connected to Ath1 function (Harris and Cotter 1988; Alizadeh and Klionsky 1996; Jules *et al.* 2004; Parrou *et al.* 2005; Huang *et al.* 2007). It is noteworthy that both studies employed fluorescent protein fusions to Ath1, as these strategies can lead to potential confounding artifacts. For example, GFP protein fusions causing mislocalization to the vacuolar lumen or YFP protein fusions forming nonnative structures (though the same study demonstrated that this was not an issue with an mCherry protein fusion) (Roberts *et al.* 1992; Swulius and Jensen 2012). These observations suggest caution should be applied to protein localization or quantification of localization based only on 1 type of data. Further, both Ath1 localization studies localized Ath1 during growth on glucose, a condition in which proper Ath1 localization for extracellular trehalose mobilization is likely unimportant (Huang *et al.* 2007; He *et al.* 2009).

**Fig. 4.**
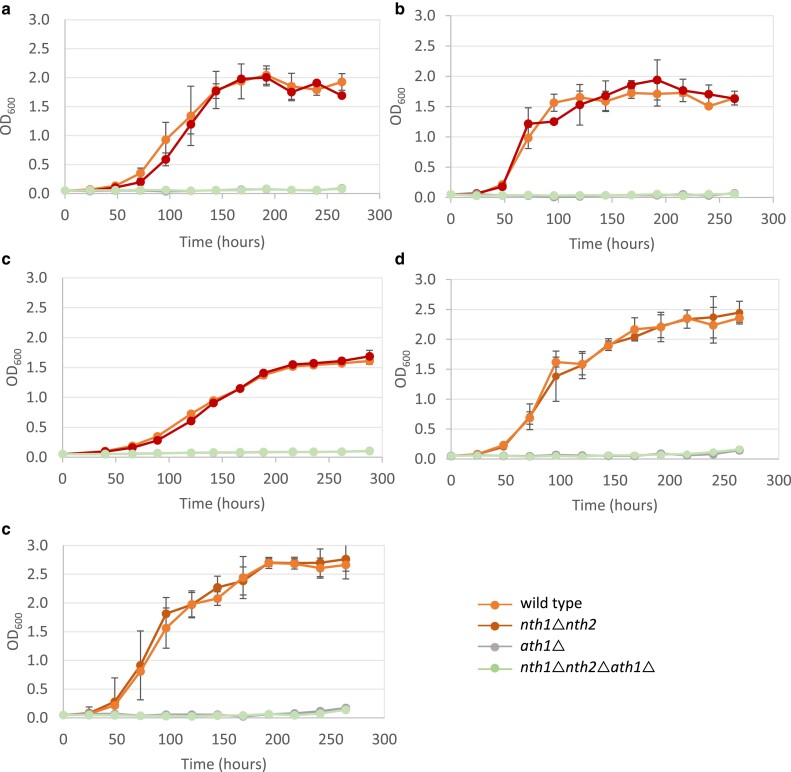
Extracellular trehalose utilization via Ath1. Growth of wild type, nth1Δ nth2Δ, ath1Δ, and nth1Δ nth2Δ ath1Δ mutants in trehalose: S288C a), Simi White b), YPS1000 c), CSM d), and Bb32 e). Cells were first inoculated into YNB + 2% glucose, incubated overnight, washed once with water, and subcultured to OD_600_ of 0.05 into YNB + 1% trehalose. OD_600_ was recorded every day for 12 days. Three biological replicates were performed, and the values are presented as the mean ± SD.

**Fig. 5.**
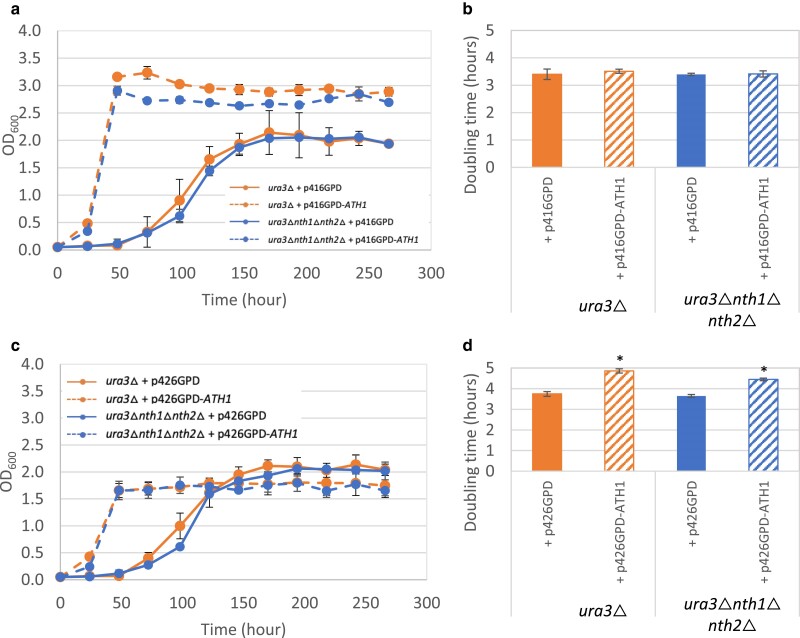
Plasmid-based ATH1 overexpression increases the rate of extracellular trehalose utilization. Growth of wild-type and trehalase mutants with and without ATH1 overexpressed from a low-copy a and b) or high-copy c and d) plasmid. a and c) Cells were grown to stationary phase in YNB + 2% glucose, washed once with sterile water, and subcultured to OD_600_ of 0.05 in YNB + 1% trehalose followed by daily measurement of cell concentration. b and d) Cells were grown to stationary phase in YNB + 2% glucose before subcultured to OD_600_ of 0.05 in YPD in a 96-well plate, which was then incubated at 30 °C for 48 h. Growth curves were used to calculate doubling time during exponential growth. Three biological replicates were performed for each experiment, and the values are presented as the mean ± SD. Asterisks represent statistically significant differences (*P* < 0.05) between the mutants and their isogenic control strains.

As multiple previous studies have demonstrated that Ath1 is required for extracellular trehalose degradation, we first sought to evaluate whether this holds true for the nonlaboratory strains tested here (Alizadeh and Klionsky 1996; Nwaka *et al.* 1996; Jules *et al.* 2004). We included deletion of the *NTH* genes, *nth1*Δ *nth2*Δ, with or without deletion of *ATH1* for comparison with strains lacking either all cellular trehalase enzyme activity or only lacking cytosolic trehalase activity, respectively. Both *ath1*Δ single mutants and *nth1*Δ*nth2*Δ*ath1*Δ triple mutants failed to grow on trehalose in all 5 genetic backgrounds, whereas *nth1*Δ*nth2*Δ mutants exhibited similar growth rates compared to their isogenic wild-type strains in trehalose-containing media (Fig. 4). To evaluate effects of *ATH1* overexpression on extracellular trehalose utilization, we used the S288C laboratory strain background due to an available auxotrophic mutation that prevents uracil biosynthesis, *ura3*Δ*0*, which was subsequently used in all experiments requiring plasmid selection. We also examined yeast cells overexpressing *ATH1* from a low- or high-copy plasmid under control of the strong, constitutive *TDH3* promoter (Mumberg *et al.* 1994). We found that low-copy overexpression of *ATH1* (p416GPD-*ATH1*) in both wild type and *nth1*Δ *nth2*Δ decreased lag phase and increased growth rate in a minimal medium containing trehalose as the sole carbon source, highlighting the sufficiency of Ath1 for extracellular trehalose mobilization (Fig. 5a). This result also demonstrates that the presence or absence of Nth1/2 activity had no effect on extracellular trehalose utilization. Expression of *ATH1* from a low-copy plasmid (p416GPD-*ATH1*) did not have any effects on growth rate in minimal glucose medium (Fig. 5b). These observations align with a previous study that observed constitutive overexpression of *ATH1* under the control of the *TDH3* promoter increased acid trehalase activity 4-fold in intact cells, with a 3-fold reduction of time in lag phase (Jules *et al.* 2004). *ATH1* overexpression from a high-copy plasmid (p426GPD-*ATH1*) resulted in similar lag phase reduction and increased growth rate, though expression at high-copy levels also had negative consequences for cellular physiology, including lower stationary phase cell concentrations and a slower growth rate in standard minimal glucose medium (Fig. 5c and d). One potential interpretation of this phenomenon is protein burden, which has been observed in overexpression systems of multiple glycolytic proteins (Vind *et al.* 1993; Gong *et al.* 2006). It is also possible that as a membrane-localized protein, excessive Ath1 expression levels could decrease the efficiency or function of the protein secretion machinery or membrane functionality, ultimately leading to a growth defect. Together, these results corroborate the necessary and sufficient role of Ath1 in extracellular trehalose utilization, along with increased Ath1 activity associated with its overexpression.

**Fig. 6.**
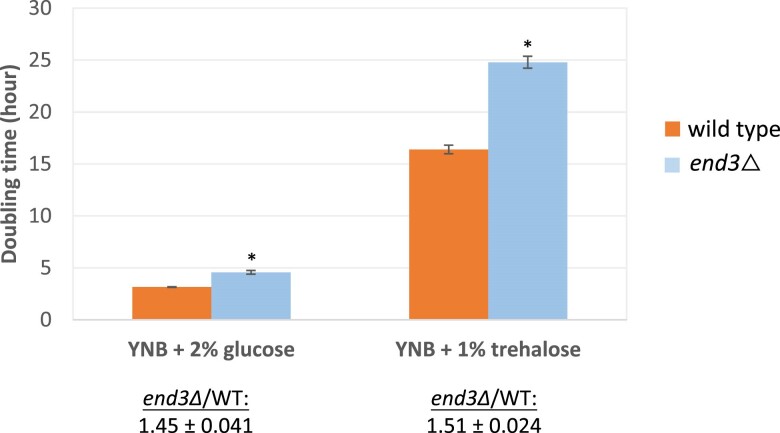
Comparison of doubling times between wild type and end3Δ mutant in glucose and trehalose. Cells were grown to stationary phase in YNB + 2% glucose before being diluted into fresh YNB + 2% glucose or YNB + 1% trehalose liquid media to an initial OD_600_ of 0.05 in 96-well microplates. The plates were then shaken at 30 °C, and the absorbance of each well was measured every 15 min (48 h in total) in double orbital shaking mode at a speed of 559 cpm. Three biological replicates were performed to calculate exponential doubling times, and the values are presented as the mean ± SD. Asterisks represent statistically significant differences (*P* < 0.05) between the mutants and their isogenic control strains. Note that the average doubling time ratio (end3Δ/WT) from the 3 replicates is reported below each carbon source; the 2 doubling time ratios are not significantly different based on a *t*-test with a significance threshold of *P* < 0.05.

However, these results do not inform whether or not trehalose is consumed by extracellular hydrolysis to glucose or import and hydrolysis in the vacuole. If consumption of extracellular trehalose occurs through import of trehalose into cells and then into the vacuole for subsequent hydrolysis to glucose by Ath1, then one possibility for trehalose import and transport to the vacuole is endocytosis. One study mentioned that an endocytosis mutant failed to grow on trehalose, potentially supporting this model, though no data were shown (Nwaka *et al.* 1996). Therefore, to test this hypothesis, we constructed an *end3*Δ mutant, which is reportedly defective in receptor-mediated and fluid-phase endocytosis (Raths *et al.* 1993). Despite its decreased endocytic activity, *end3*Δ had a similar growth defect on trehalose compared to its growth defect on glucose: roughly 50% slower than wild type on either carbon source (Fig. 6). This suggests that endocytosis is not a major mechanism of trehalose import, and no alternative model for extracellular trehalose transport to the vacuole has been proposed. This result supports the alternative model described previously in which extracellular trehalose is metabolized to glucose by extracellular Ath1 tethered to the plasma membrane (Jules *et al.* 2004; He *et al.* 2009). However, we and others have observed an increase in intracellular trehalose in an *ath1*Δ strain, which may represent accumulated trehalose in the vacuole, though the cellular role or significance of vacuolar-localized trehalose is unclear (Fig. 2) (Garre *et al.* 2009).

### The role of Agt1 in extracellular trehalose utilization

In addition to Ath1, some *S. cerevisiae* strains possess an alternate path for consuming extracellular trehalose: Agt1-mediated trehalose transport into the cytoplasm followed by intracellular Nth1/2-mediated hydrolysis (Jules *et al.* 2004; Gibney *et al.* 2015). The Agt1 transporter is a proton-disaccharide symporter also known as Mal11. Agt1 has accumulated mutations conferring a broader substrate range than its ancestral maltose transporter and can transport trehalose among other disaccharides (Fig. 1) (Han *et al.* 1995; Brown *et al.* 2010; Gibney *et al.* 2015). The observation that deletion of *ATH1* abrogates growth on trehalose in all tested strains implies that trehalose import through the Atg1 transport protein may not be a common, natural alternate route for trehalose import. To further investigate this possibility, we first designed primers to selectively amplify a fragment *AGT1* (Supplementary Fig. 5). PCR amplification and gel electrophoresis revealed that S288C, YPS1000, and Bb32 contain *AGT1*, while Simi White and CSM do not (Supplementary Fig. 5). As *AGT1* belongs to the *MAL* gene family, we hypothesized that preculturing on maltose might induce native Agt1 expression, enabling growth on trehalose despite the absence of *ATH1*; pregrowth in maltose to increase trehalose utilization has been previously described (François and Parrou 2001). Therefore, we first evaluated whether these strains contain a maltose transport gene and the ability to grow in maltose. *MAL31*, another gene in the MAL family, encodes a transport protein specific to maltose. While PCR confirmed that all 5 tested strains contain *MAL31*, S288C and YPS1000 did not grow in maltose (Supplementary Fig. 5). S288C-derived strains encode a mutated, inactive maltose regulator protein, resulting in failure to express any *MAL* gene and concomitant inability to grow on maltose (Supplementary Fig. 5) (Brown *et al.* 2010). Consequently, we were only able to pregrow Simi White, Bb32, and CSM in maltose (though Bb32 is the only 1 of these 3 containing *AGT1*), while S288C and YPS1000 were pregrown in glucose. While all wild-type strains were able to grow in minimal trehalose medium, none of the *ath1*Δ mutants exhibited growth under the same conditions, regardless of pregrowth regime (Supplementary Fig. 5e). As a positive control, we compared the growth of *ath1*Δ strains with an *ath1*Δ mutant strain overexpressing Agt1 from the S288C genetic background (Gibney *et al.* 2015). As expected, based on previous literature, overexpressed Agt1 restored growth on trehalose, recapitulating the observation that the Agt1 transporter allows growth in trehalose when *ATH1* is absent (Supplementary Fig. 5e) (Jules *et al.* 2004; Gibney *et al.* 2015). In our experiment, the only tested strain able to pregrow on maltose that also encodes *AGT1* was Bb32. As Bb32 *ath1*Δ was also unable to grow using extracellular trehalose regardless of pregrowth regime, it is possible that either Bb32's *AGT1* allele contains loss-of-function mutations, or that it is not expressed in the conditions tested potentially due to promoter mutation or because it is part of a silenced copy of the MAL regulon in the subtelomeric region of the chromosome (Brown *et al.* 2010). Based on these results, while *AGT1* can clearly be used to transport extracellular trehalose when constitutively overexpressed, it is unclear how often native expression of *AGT1* supports growth on extracellular trehalose. Evaluation of further strains will be required to demonstrate a role for native expression of *AGT1* in extracellular trehalose utilization.

### Intracellular trehalose utilization during lag phase

**Fig. 7.**
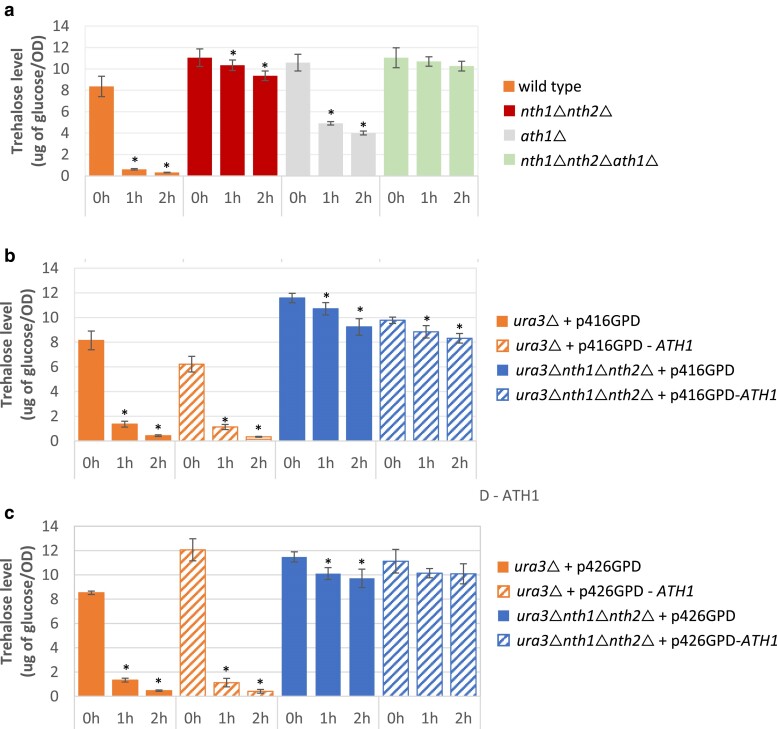
Intracellular trehalose utilization during lag phase. a–c) Stationary phase cells (0 h) from indicated strains pregrown overnight in YNB + 2% glucose were diluted into fresh YNB +2% glucose liquid media with an OD_600_ of 0.5 and then incubated at 30 °C for 1 hour (1 h) and 2 hours (2 h) before samples were taken for trehalose content measurement. Three biological replicates were performed, and the values are presented as the mean with SD. Asterisks represent statistically significant differences (*P* < 0.05) between an individual time point compared to its respective time 0.

Slow growing or nongrowing cells produce and accumulate high levels of intracellular trehalose, up to 15–20% of dry cell weight, which is quickly mobilized after addition of sufficient nutrients (Silljé *et al.* 1999). To further examine the role of trehalase enzymes in intracellular trehalose degradation, we monitored the change in intracellular trehalose levels during lag phase in wild-type cells and multiple trehalase mutants from the S288C genetic background. When we transferred stationary phase cells to fresh growth media, we observed that most of the intracellular trehalose in wild-type cells was hydrolyzed within 1–2 h (Fig. 7a). By taking time points at 1 and 2 h after exposure to fresh media, we expect that reduction in trehalose levels represents intracellular enzymatic trehalose degradation rather than dilution by cell division. As anticipated, when both *NTH1* and *NTH2* were deleted (*nth1*Δ*nth2*Δ), the initial concentration of intracellular trehalose and rate of trehalose degradation was much slower compared to wild type. In the *ath1*Δ mutant, intracellular trehalose was still consumed, though not as completely as observed for wild-type cells. In the triple trehalase gene deletion strain (*nth1*Δ*nth2*Δ*ath1*Δ), intracellular trehalose levels did not change significantly between tested time points. This is consistent with previous findings by Parrou *et al.* (2005) that demonstrated that an *nth1nth2* mutant could still mobilize trehalose during prolonged incubation in the stationary phase, but further deletion of *ATH1* completely abolished this mobilization (Parrou *et al.* 2005). These results align with the notion that the majority of intracellular trehalose degradation relies on the Nth1/2 enzymes, with Ath1 also playing some role in intracellular trehalose utilization, potentially related to vacuolar localization.

We also evaluated the effect of *ATH1* overexpression on the degradation rate of intracellular trehalose during lag phase. Plasmid-based *ATH1* expression was insufficient to degrade intracellular trehalose to wild-type levels when the Nth1/2 enzymes were absent (Fig. 7b and c). This suggests that increased Ath1 activity associated with overexpression is unable to hydrolyze bulk cytoplasmic trehalose, a function performed by Nth1/2 enzymes, which also aligns with their pH optima. However, unconsumed trehalose in *ath1*Δ mutants during lag phase indicates that a fraction of Ath1-susceptible intracellular trehalose produced in the cytoplasm by glucose-grown stationary phase cells can accumulate in a cellular compartment not accessible to or functionally compatible with Nth1/2, likely the vacuole. While there is no evidence that extracellular trehalose is transported into the vacuole as a utilization route, these results suggest that intracellular trehalose can enter the vacuole, though the mechanism of transport is not characterized. Together, these results suggest that Ath1 degrades vacuolar-localized trehalose, though the biological significance of vacuolar trehalose accumulation/degradation remains unclear and unrelated to the role of Ath1 in extracellular trehalose utilization. Overall, these results indicate that intracellular cytoplasmic trehalose is primarily hydrolyzed by Nth1/2, though Ath1 also has a minor role in intracellular trehalose hydrolysis, likely inside the vacuole.

### Intracellular trehalose utilization during spore germination

**Fig. 8.**
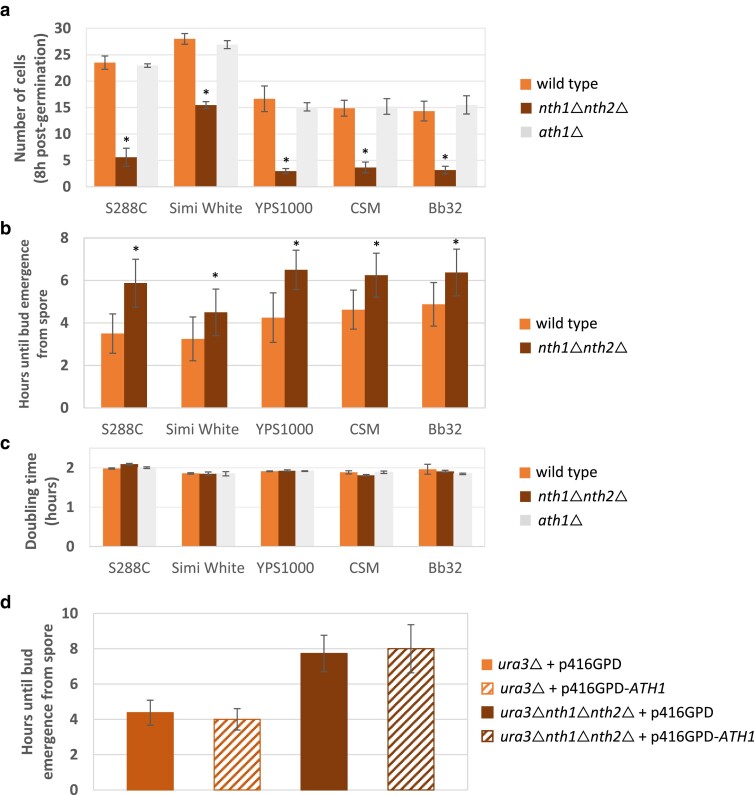
Absence of neutral trehalases results in a spore germination defect. a) Cells were grown overnight in YPD before being sporulated for 6 days at room temperature. Six tetrads for each strain were dissected onto YPD plates, and the number of cells grown from each germinated spore was counted after an 8-h incubation at 30 °C. b) Two tetrads (8 spores) from each sporulated cultures were dissected on YPD plates and incubated at 30 °C. Through visual inspection each hour, the number of hours required until bud emergence was recorded. c) Doubling times for indicated strains in YPD were calculated from plate reader-based growth curves as described in *Materials and methods*. d) Six tetrads (24 spores) from 3 independent sporulated cultures were dissected on YPD plates and incubated at 30 °C. Bud emergence time was evaluated as above. Values are presented as the mean with SD. Asterisks represent statistically significant differences (*P* < 0.05) between the mutants and their isogenic wild-type strains.

Sporulation in the budding yeast *S. cerevisiae* involves meiotic cell division followed by formation of 4 haploid spores containing high levels of trehalose (Geijer *et al.* 2012). Upon exposure to glucose and other nutrients, spores exit dormancy through germination and resume growth. Spore germination of *S. cerevisiae* is a multistep developmental path in which trehalose is rapidly mobilized (Rousseau *et al.* 1972). Therefore, we sought to evaluate the role of intracellular trehalose in spore germination. Germination was evaluated after tetrads were dissected on glucose-containing rich media. All 5 tested *nth1*Δ*nth2*Δ mutants exhibited significantly slower germination than their isogenic parent and respective *ath1*Δ mutant (Fig. 8a). This germination defect was associated with the first cell division, as all evaluated *nth1*Δ*nth2*Δ mutants displayed significantly slower emergence of the first cell compared to their isogenic wild-type parent (Fig. 8b). There was no effect on subsequent exponential growth rate, or, presumably, cell cycle progression, as all the trehalase mutants had statistically indistinguishable doubling times compared to their isogenic wild-type parent (Fig. 8c). Similar results were observed in the fission yeast *Schizosaccharomyces pombe* when spores from strains lacking neutral trehalase (*ntp1*-) were induced to germinate: the onset of this process was markedly delayed and germination rate was reduced (Beltran *et al.* 2000). To determine whether this was a germination-specific defect or a general lag phase defect associated with dormant cells, we also examined single-cell lag phase times for quiescent trehalase mutants (*nth1*Δ*nth2*Δ and *ath1*Δ) and observed no lag phase extension (Supplementary Fig. 6). These results indicate that intracellular cytoplasmic trehalose hydrolysis has an important role in promoting exit from spore dormancy, though not in exiting the quiescent state. None of the *ath1*Δ spores or quiescent cells exhibited slower cell division upon exposure to nutrients, and all exhibited the same logarithmic growth rate as their isogenic wild types in glucose media, indicating that failure to degrade vacuolar trehalose, if present, has no effect on spore germination or postquiescent lag phase (Fig. 8a and c). It seems likely that the germination defect in neutral trehalase mutants results from decreased available trehalose-derived glucose, suggesting an important role for trehalose as a storage carbohydrate specifically during sporulation, though not during quiescence. This result contrasts with that described by Shi *et al.* (2010), where wild-type CEN.PK cells were able to recover from quiescence faster if they contained more intracellular trehalose (pregrowth in rich medium) compared to cells with less intracellular trehalose (pregrowth in minimal medium). While Shi *et al*. indicates a correlation between intracellular trehalase content and lag phase duration postquiescence, a number of other explanations could also explain the result, including that the laboratory CEN.PK strain lineage contains a mutation in adenylate cyclase (*cyr1*-K1867M) that affects nutrient sensing and signaling, or variable survivability in rich vs minimal medium (Vanhalewyn *et al.* 1999; Dumortier *et al.* 2000).

Previous studies have suggested the possibility that intracellular trehalose accumulation may be important for cell cycle regulation. As G1/G0 cells accumulate intracellular trehalose and hydrolyze it upon cell cycle start, it has been proposed that trehalose is important for entering the cell cycle, though no cell cycle defects have been observed for trehalose metabolism mutants growing in standard growth media conditions (Küenzi and Fiechter 1969; Silljé *et al.* 1999; Futcher 2006; Chen *et al.* 2007; Shi *et al.* 2010). In this conceptual model, cells require glucose from hydrolyzed trehalose as the glycolytic fuel to initiate the cell cycle. In support of this notion, trehalose and glycogen hydrolyzing enzymes are regulated by cell cycle regulators, and cells lacking both neutral trehalase and glycogen phosphorylase activity (*nth1*Δ*nth2*Δ*gph1*Δ) exhibited significant changes in the dynamics of central carbon metabolites and amino acid metabolism during cell cycle progression, alongside a complete loss of sporulation ability (Ewald *et al*. 2016; Zhao *et al*. 2016). In the same studies, Nth1 was activated at the G1/S transition, which would funnel trehalose-derived glucose into glycolysis during S phase; this activation was proposed to support cell cycle progression in poor nutrient environments. In accordance with this observation, Ewald *et al*. (2016) demonstrated that cells unable to hydrolyze trehalose and glycogen (*nth1*Δ*nth2*Δ*gph1*Δ) had a lower probability of completing a cell cycle after shifting from 0.005% w/v glucose to 0% glucose in a microfluidic device. This phenotype was reversed when Nth1 function was restored (Ewald *et al*. 2016). The spore germination defect observed associated with all *nth1*Δ*nth2*Δ strains tested in this study demonstrates another cell cycle progression defect associated with failure to hydrolyze trehalose.

The germination defect associated with *nth1*Δ*nth2*Δ also provides an opportunity to further test whether Ath1 has a role in liberating glucose from intracellular trehalose to support cellular growth. If *ATH1* overexpression is able to suppress the germination defect associated with *nth1*Δ*nth2*Δ, this would be evidence for a functional role associated with Ath1-based hydrolysis of intracellular trehalose. However, overexpression of *ATH1* did not decrease the amount of time required for bud emergence from *nth1*Δ*nth2*Δ spores (Fig. 8d). This result further suggests that Ath1 does not appear to have a meaningful intracellular role in producing glucose from intracellular trehalose. It is noteworthy that in this experiment, 8 of the 48 tested *nth1*Δ*nth2*Δ spores completely failed to germinate, compared with 1 of the 48 tested wild-type spores (Supplementary Fig. 7). This suggests that the increased germination lag phase observed in these mutants can sometimes result in a complete germination failure, though the mechanism determining these variable outcomes remains elusive. Similarly, Ewald *et al*. (2016) demonstrated that mutants unable to hydrolyze trehalose and glycogen (*nth1*Δ*nth2*Δ*gph1*Δ) had a significantly higher rate of cell divisions resulting in nonviable cells during starvation compared to wild type, ∼15% vs ∼3%.

### Conceptual model for trehalose degradation in *S. cerevisiae*

**Fig. 9.**
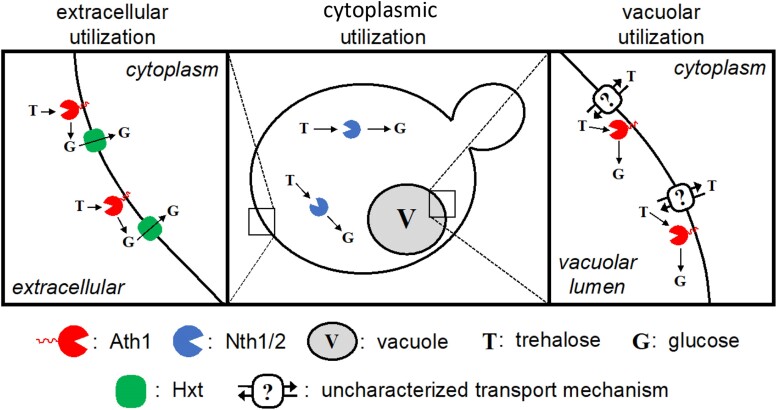
Conceptual model illustrating multiple modes of trehalose degradation in *S. cerevisiae*. Left: extracellular utilization of trehalose as a carbon source requires extracellular degradation of trehalose into glucose via the Ath1 trehalase enzyme. While the Agt1/Mal11 protein (not pictured) can also import extracellular trehalose when overexpressed, it remains unclear whether it is expressed/utilized for trehalose consumption in wild strains. Center: cytoplasmic trehalose is degraded into glucose via the Nth1 and Nth2 trehalase enzymes, an activity required for proper spore germination. Right: while of unclear functional significance, vacuolar trehalose enters the vacuole through an uncharacterized mechanism and is degraded by the Ath1 trehalase enzyme.

In Fig. 9, we have produced a conceptual model for intracellular and extracellular trehalose utilization in *S. cerevisiae*. Previously published information along with data presented here support the notion that extracellular trehalose is primarily utilized through the action of extracellular Ath1. This enzyme can hydrolyze trehalose into glucose, which is then imported and can be used for glycolysis. Further, while the Agt1 protein has the ability to transport trehalose when overexpressed, it remains unclear how often this route of trehalose import/utilization is used instead of Ath1-based hydrolysis, especially when *AGT1* is under the regulation of its native promoter. Previously published information along with data presented here support the notion that cytosolic trehalose is primarily utilized through the action of Nth1/2. We also demonstrated that Nth1/2-based trehalose mobilization is required to support rapid germination from the spore state. Overexpression of *ATH1* was unable to completely hydrolyze intracellular trehalose during lag phase and failed to suppress the *nth1*Δ*nth2*Δ germination defect, further suggesting that Ath1 does not typically function to release glucose from intracellular trehalose. This is in line with its reported plasma membrane and vacuolar localization, in addition to its acidic pH optimum. Finally, we suggest that the fraction of intracellular trehalose present in *ath1*Δ mutants represents vacuolar trehalose. While it appears that vacuolar Ath1 can hydrolyze vacuolar trehalose, the functional consequences of this activity are an open question. Similarly, it remains to be seen whether Ath1 vacuolar localization is important for cellular physiology or if it is simply an artifact associated with plasma membrane recycling.

## Supplementary Material

jkae215_Supplementary_Data

## Data Availability

Strains and plasmids are available upon request. The authors affirm that all data necessary for confirming the conclusions of the article are present within the article, figures, and tables. Supplemental material available at G3 online.

## References

[jkae215-B1] Alblova M , Smidova A, Kalabova D, Lentini Santo D, Obsil T, Obsilova V. 2019. Allosteric activation of yeast enzyme neutral trehalase by calcium and 14-3-3 protein. Physiol Res. 68(2):147–160. doi:10.33549/physiolres.933950. 30628830

[jkae215-B2] Alizadeh P , Klionsky DJ. 1996. Purification and biochemical characterization of the ATH1 gene product, vacuolar acid trehalase, from *Saccharomyces cerevisiae*. FEBS Lett. 391(3):273–278. doi:10.1016/0014-5793(96)00751-X. 8764988

[jkae215-B3] Beltran FF , Castillo R, Vicente-Soler J, Cansado J, Gacto M. 2000. Role for trehalase during germination of spores in the fission yeast *Schizosaccharomyces pombe*. FEMS Microbiol Lett. 193(1):117–121. doi:10.1111/j.1574-6968.2000.tb09412.x. 11094289

[jkae215-B4] Brown CA , Murray AW, Verstrepen KJ. 2010. Rapid expansion and functional divergence of subtelomeric gene families in yeasts. Curr Biol. 20(10):895–903. doi:10.1016/j.cub.2010.04.027. 20471265 PMC2877759

[jkae215-B5] Chen A , Gibney PA. 2022. Intracellular trehalose accumulation via the Agt1 transporter promotes freeze–thaw tolerance in *Saccharomyces cerevisiae*. J Appl Microbiol. 133(4):2390–2402. doi:10.1111/jam.15700. 35801661

[jkae215-B6] Chen A , Vargas-Smith J, Tapia H, Gibney PA. 2022. Characterizing phenotypic diversity of trehalose biosynthesis mutants in multiple wild strains of *Saccharomyces cerevisiae*. G3 (Bethesda). 12(11):jkac196. doi:10.1093/g3journal/jkac196. 35929793 PMC9635654

[jkae215-B7] Chen Z , Odstrcil EA, Tu BP, McKnight SL. 2007. Restriction of DNA replication to the reductive phase of the metabolic cycle protects genome integrity. Science. 316(5833):1916–1919. doi:10.1126/science.1140958. 17600220

[jkae215-B8] Chopra R , Sharma VM, Ganesan K. 1999. Elevated growth of *Saccharomyces cerevisiae* ATH1 null mutants on glucose is an artifact of nonmatching auxotrophies of mutant and reference strains. Appl Environ Microbiol. 65(5):2267–2268. doi:10.1128/AEM.65.5.2267-2268.1999. 10224035 PMC91332

[jkae215-B9] Crowe JH , Crowe LM, Chapman D. 1984. Preservation of membranes in anhydrobiotic organisms: the role of trehalose. Science. 223(4637):701–703. doi:10.1126/science.223.4637.701. 17841031

[jkae215-B10] de Silva-Udawatta MN , Cannon JF. 2001. Roles of trehalose phosphate synthase in yeast glycogen metabolism and sporulation. Mol Microbiol. 40(6):1345–1356. doi:10.1046/j.1365-2958.2001.02477.x. 11442833

[jkae215-B11] Destruelle M , Holzer H, Klionsky DJ. 1995. Isolation and characterization of a novel yeast gene, ATH1, that is required for vacuolar acid trehalase activity. Yeast. 11(11):1015–1025. doi:10.1002/yea.320111103. 7502577

[jkae215-B12] Duina AA , Miller ME, Keeney JB. 2014. Budding yeast for budding geneticists: a primer on the *Saccharomyces cerevisiae* model system. Genetics. 197(1):33–48. doi:10.1534/genetics.114.163188. 24807111 PMC4012490

[jkae215-B13] Dumortier F , Vanhalewyn M, Debast G, Colombo S, Ma P, Winderickx J, Van Dijck P, Thevelein JM. 2000. A specific mutation in *Saccharomyces cerevisiae* adenylate cyclase, Cyr1(K1876M), eliminates glucose- and acidification-induced cAMP signalling and delays glucose-induced loss of stress resistance. Int J Food Microbiol. 55(1–3):103–107. doi:10.1016/S0168-1605(00)00184-7. 10791726

[jkae215-B14] Elbein AD , Pan YT, Pastuszak I, Carroll D. 2003. New insights on trehalose: a multifunctional molecule. Glycobiology. 13(4):17R–27R. doi:10.1093/glycob/cwg047. 12626396

[jkae215-B15] Engel SR , Dietrich FS, Fisk DG, Binkley G, Balakrishnan R, Costanzo MC, Dwight SS, Hitz BC, Karra K, Nash RS, et al 2014. The reference genome sequence of *Saccharomyces cerevisiae*: then and now. G3 (Bethesda). 4(3):389–398. doi:10.1534/g3.113.008995. 24374639 PMC3962479

[jkae215-B16] Ewald JC , Kuehne A, Zamboni N, Skotheim JM. 2016. The yeast cyclin-dependent kinase routes carbon fluxes to fuel cell cycle progression. Mol Cell. 62(4):532–545. doi:10.1016/j.molcel.2016.02.017. 27203178 PMC4875507

[jkae215-B17] François J , Neves MJ, Hers HG. 1991. The control of trehalose biosynthesis in *Saccharomyces cerevisiae*: evidence for a catabolite inactivation and repression of trehalose-6-phosphate synthase and trehalose-6-phosphate phosphatase. Yeast. 7(6):575–587. doi:10.1002/yea.320070605. 1662849

[jkae215-B18] François J , Parrou JL. 2001. Reserve carbohydrates metabolism in the yeast *Saccharomyces cerevisiae*. FEMS Microbiol Rev. 25(1):125–145. doi:10.1111/j.1574-6976.2001.tb00574.x. 11152943

[jkae215-B19] Futcher B . 2006. Metabolic cycle, cell cycle, and the finishing kick to start. Genome Biol. 7(4):107. doi:10.1186/gb-2006-7-4-107. 16677426 PMC1557985

[jkae215-B20] Gancedo C , Flores CL. 2004. The importance of a functional trehalose biosynthetic pathway for the life of yeasts and fungi. FEMS Yeast Res. 4(4–5):351–359. doi:10.1016/S1567-1356(03)00222-8. 14734015

[jkae215-B21] Garre E , Pérez-Torrado R, Gimeno-Alcañiz JV, Matallana E. 2009. Acid trehalase is involved in intracellular trehalose mobilization during postdiauxic growth and severe saline stress in *Saccharomyces cerevisiae*. FEMS Yeast Res. 9(1):52–62. doi:10.1111/j.1567-1364.2008.00453.x. 19016884

[jkae215-B22] Geijer C , Pirkov I, Vongsangnak W, Ericsson A, Nielsen J, Krantz M, Hohmann S. 2012. Time course gene expression profiling of yeast spore germination reveals a network of transcription factors orchestrating the global response. BMC Genomics. 13:554. doi:10.1186/1471-2164-13-554. 23066959 PMC3577491

[jkae215-B23] Giaever G , Chu AM, Ni L, Connelly C, Riles L, Véronneau S, Dow S, Lucau-Danila A, Anderson K, André B, et al 2002. Functional profiling of the *Saccharomyces cerevisiae* genome. Nature. 418(6896):387–391. doi:10.1038/nature00935. 12140549

[jkae215-B24] Gibney PA , Schieler A, Chen JC, Rabinowitz JD, Botstein D. 2015. Characterizing the in vivo role of trehalose in *Saccharomyces cerevisiae* using the AGT1 transporter. Proc Natl Acad Sci U S A. 112(119):6116–6121. doi:10.1073/pnas.1506289112. 25918382 PMC4434743

[jkae215-B25] Gibson DG , Young L, Chuang R-Y, Venter JC, Hutchison CA, Smith HO. 2009. Enzymatic assembly of DNA molecules up to several hundred kilobases. Nat Methods. 6(5):343–345. doi:10.1038/nmeth.1318. 19363495

[jkae215-B26] Goffeau A , Barrell G, Bussey H, Davis RW, Dujon B, Feldmann H, Galibert F, Hoheisel JD, Jacq C, Johnston M, et al 1996. Life with 6000 genes. Science. 274(5287):546–567. doi:10.1126/science.274.5287.546. 8849441

[jkae215-B27] Gong M , Gong F, Yanofsky C. 2006. Overexpression of tnaC of *Escherichia coli* inhibits growth by depleting tRNA2Pro availability. J Bacteriol. 188(5):1892–1898. doi:10.1128/JB.188.5.1892-1898.2006. 16484200 PMC1426567

[jkae215-B28] González MI , Blázquez MA, Gancedo C, Stucka R, Feldmann H. 1992. Molecular cloning of CIF1, a yeast gene necessary for growth on glucose. Yeast. 8(3):183–192. doi:10.1002/yea.320080304. 1315471

[jkae215-B29] Guthrie C , Fink GR. 1991. Guide to Yeast Genetics and Molecular Biology. 1st ed. (Methods in Enzymology, vol. 194). Academic Press. p. 1–863. 2005781

[jkae215-B30] Han EK , Cotty F, Sottas C, Jiang H, Michels CA. 1995. Characterization of AGT1 encoding a general α-glucoside transporter from *Saccharomyces*. Mol Microbiol. 17(6):1093–1107. doi:10.1111/j.1365-2958.1995.mmi_17061093.x. 8594329

[jkae215-B31] Harris SD , Cotter DA. 1988. Transport of yeast vacuolar trehalase to the vacuole. Can J Microbiol. 34(7):835–838. doi:10.1139/m88-143. 3058274

[jkae215-B32] He S , Bystricky K, Leon S, François JM, Parrou JL. 2009. The *Saccharomyces cerevisiae* vacuolar acid trehalase is targeted at the cell surface for its physiological function. FEBS J. 276(19):5432–5446. doi:10.1111/j.1742-4658.2009.07227.x. 19703229

[jkae215-B33] Hottiger T , Boller T, Wiemken A. 1987. Rapid changes of heat and desiccation tolerance correlated with changes of trehalose content in *Saccharomyces cerevisiae* cells subjected to temperature shifts. FEBS Lett. 220(1):113–115. doi:10.1016/0014-5793(87)80886-4. 3301407

[jkae215-B34] Hounsa C-G , Brandt EV, Thevelein J, Hohmann S, Prior BA. 1998. Role of trehalose in survival of *Saccharomyces cerevisiae* under osmotic stress. Microbiology. 144:671–680. doi:10.1099/00221287-144-3-671. 9534237

[jkae215-B35] Huang J , Reggiori F, Klionsky DJ. 2007. The transmembrane domain of acid trehalase mediates ubiquitin-independent multivesicular body pathway sorting. Mol Biol Cell. 18(7):2511–2524. doi:10.1091/mbc.e06-11-0995. 17475771 PMC1924822

[jkae215-B36] Huh W-K , Falvo JV, Gerke LC, Carroll AS, Howson RW, Weissman JS, O'Shea EK. 2003. Global analysis of protein localization in budding yeast. Nature. 425(6959):686–691. doi:10.1038/nature02026. 14562095

[jkae215-B37] Jules M , Beltran G, François J, Parrou JL. 2008. New insights into trehalose metabolism by *Saccharomyces cerevisiae*: NTH2 encodes a functional cytosolic trehalase, and deletion of TPS1 reveals Ath1p-dependent trehalose mobilization. Appl Environ Microbiol. 74(3):605–614. doi:10.1128/AEM.00557-07. 18065618 PMC2227697

[jkae215-B38] Jules M , Guillou V, François J, Parrou JL. 2004. Two distinct pathways for trehalose assimilation in the yeast *Saccharomyces cerevisiae*. Appl Environ Microbiol. 70(5):2771–2778. doi:10.1128/AEM.70.5.2771-2778.2004. 15128531 PMC404389

[jkae215-B39] Kim J , Alizadeh P, Harding T, Hefner-Gravink A, Klionsky DJ. 1996. Disruption of the yeast ATH1 gene confers better survival after dehydration, freezing, and ethanol shock: potential commercial applications. Appl Environ Microbiol. 62(5):1563–1569. doi:10.1128/aem.62.5.1563-1569.1996. 8633854 PMC167930

[jkae215-B40] Küenzi MT , Fiechter A. 1969. Changes in carbohydrate composition and trehalase-activity during the budding cycle of *Saccharomyces cerevisiae*. Archiv für Mikrobiologie. 64(4):396–407. doi:10.1007/BF00417021. 4916776

[jkae215-B41] Lillie SH , Pringle JR. 1980. Reserve carbohydrate metabolism in *Saccharomyces cerevisiae*: responses to nutrient limitation. J Bacteriol. 143(3):1384–1394. doi:10.1128/jb.143.3.1384-1394.1980. 6997270 PMC294518

[jkae215-B42] Mortimer RK , Johnston JR. 1986. Genealogy of principal strains of the yeast genetic stock center. Genetics. 113(1):35–43. doi:10.1093/genetics/113.1.35. 3519363 PMC1202798

[jkae215-B43] Mumberg D , Muller R, Funk M. 1994. Regulatable promoters of *Saccharomyces cerevisiae*: comparison of transcriptional activity and their use for heterologous expression. Nucleic Acids Res. 22(25):5767–5768. doi:10.1093/nar/22.25.5767. 7838736 PMC310147

[jkae215-B44] Mumberg D , Müller R, Funk M. 1995. Yeast vectors for the controlled expression of heterologous proteins in different genetic backgrounds. Gene. 156(1):119–122. doi:10.1016/0378-1119(95)00037-7. 7737504

[jkae215-B45] Navon G , Shulman RG, Yamane T, Eccleshall TR, Lam K-B, Baronofsky Jerald J., Marmur Julius. 1979. Phosphorus-31 nuclear magnetic resonance studies of wild-type and glycolytic pathway mutants of *Saccharomyces cerevisiae*. Biochemistry. 18(21):4487–4499. doi:10.1021/bi00588a006. 40590

[jkae215-B46] Neves MJ , Hohmann S, Bell W, Dumortier F, Luyten K, Ramos J, Cobbaert P, de Koning W, Kaneva Z, Thevelein JM. 1995. Control of glucose influx into glycolysis and pleiotropic effects studied in different isogenic sets of *Saccharomyces cerevisiae* mutants in trehalose biosynthesis. Curr Genet. 27(2):110–122. doi:10.1007/BF00313424. 7788713

[jkae215-B47] Nwaka S , Holzer H. 1997. Molecular biology of trehalose and the trehalases in the yeast *Saccharomyces cerevisiae*. Prog Nucleic Acid Res Mol Biol. 58:197–237. doi:10.1016/S0079-6603(08)60037-9. 9308367

[jkae215-B48] Nwaka S , Kopp M, Holzer H. 1995. Expression and function of the trehalase genes NTH1 and YBR0106 in *Saccharomyces cerevisiae*. J Biol Chem. 270(17):10193–10198. doi:10.1074/jbc.270.17.10193. 7730323

[jkae215-B49] Nwaka S , Mechler B, Destruelle M, Holzer H. 1995. Phenotypic features of trehalase mutants in *Saccharomyces cerevisiae*. FEBS Lett. 360(3):286–290. doi:10.1016/0014-5793(95)00105-i. 7883049

[jkae215-B50] Nwaka S , Mechler B, Holzer H. 1996. Deletion of the ATH1 gene in *Saccharomyces cerevisiae* prevents growth on trehalose. FEBS Lett. 386(2–3):235–238. doi:10.1016/0014-5793(96)00450-4. 8647289

[jkae215-B51] Olsson C , Jansson H, Swenson J. 2016. The role of trehalose for the stabilization of proteins. J Phys Chem B. 120(20):4723–4731. doi:10.1021/acs.jpcb.6b02517. 27135987

[jkae215-B52] Panni S , Landgraf C, Volkmer-Engert R, Cesareni G, Castagnoli L. 2008. Role of 14-3-3 proteins in the regulation of neutral trehalase in the yeast *Saccharomyces cerevisiae*. FEMS Yeast Res. 8(1):53–63. doi:10.1111/j.1567-1364.2007.00312.x. 17916074

[jkae215-B53] Parrou JL , François J. 1997. A simplified procedure for a rapid and reliable assay of both glycogen and trehalose in whole yeast cells. Anal Biochem. 248(1):186–188. doi:10.1006/abio.1997.2138. 9177741

[jkae215-B54] Parrou JL , Jules M, Beltran G, François J. 2005. Acid trehalase in yeasts and filamentous fungi: localization, regulation and physiological function. FEMS Yeast Res. 5(6–7):503–511. doi:10.1016/j.femsyr.2005.01.002. 15780651

[jkae215-B55] Petri JR . 1887. Eine kleine Modification des Koch'schen Plattenverfahrens (A minor modification of the pating technique of Koch). Centralblatt für Bacteriologie und Parasitenkunde. 1:279–280.

[jkae215-B56] Raths S , Rohrer J, Crausaz F, Riezman H. 1993. End3 and end4: two mutants defective in receptor-mediated and fluid-phase endocytosis in *Saccharomyces cerevisiae*. J Cell Biol. 120(1):55–65. doi:10.1083/jcb.120.1.55. 8380177 PMC2119492

[jkae215-B57] Roberts CJ , Nothwehr SF, Stevens TH. 1992. Membrane protein sorting in the yeast secretory pathway: evidence that the vacuole may be the default compartment. J Cell Biol. 119(1):69–83. doi:10.1083/jcb.119.1.69. 1527174 PMC2289628

[jkae215-B58] Rousseau P , Halvorson HO, Bulla LA, St Julian G. 1972. Germination and outgrowth of single spores of *Saccharomyces cerevisiae* viewed by scanning electron and phase-contrast microscopy. J Bacteriol. 109(3):1232–1238. doi:10.1128/jb.109.3.1232-1238.1972. 4551750 PMC247347

[jkae215-B59] San Miguel PF , Argüelles JC. 1994. Differential changes in the activity of cytosolic and vacuolar trehalases along the growth cycle of *Saccharomyces cerevisiae*. Biochim Biophys Acta. 1200(2):155–160. doi:10.1016/0304-4165(94)90130-9. 8031835

[jkae215-B60] Scott TD , Xu P, McClean MN. 2023. Strain-dependent differences in coordination of yeast signalling networks. FEBS J. 290(8):2097–2114. doi:10.1111/febs.16689. 36416575 PMC10121740

[jkae215-B61] Shao Q , Wang J, Zhu W. 2019. Trehalose stabilizing protein in a water replacement scenario: insights from molecular dynamics simulation. bioRxiv. 10.1101/2019.12.27.889063, preprint: not peer reviewed.

[jkae215-B62] Shi L , Sutter BM, Ye X, Tu BP. 2010. Trehalose is a key determinant of the quiescent metabolic state that fuels cell cycle progression upon return to growth. Mol Biol Cell. 21(12):1982–1990. doi:10.1091/mbc.e10-01-0056. 20427572 PMC2883942

[jkae215-B63] Sikorski RS , Hieter P. 1989. A system of shuttle vectors and yeast host strains designed for efficient manipulation of DNA in *Saccharomyces cerevisiae*. Genetics. 122(1):19–27. doi:10.1093/genetics/122.1.19. 2659436 PMC1203683

[jkae215-B64] Silljé HHW , Paalman JWG, Ter Schure EG, Olsthoorn SQB, Verkleij AJ, Boonstra J, Verrips CT. 1999. Function of trehalose and glycogen in cell cycle progression and cell viability in *Saccharomyces cerevisiae*. J Bacteriol. 181(2):396–400. doi:10.1128/JB.181.2.396-400.1999. 9882651 PMC93391

[jkae215-B65] Singer MA , Lindquist S. 1998a. Multiple effects of trehalose on protein folding in vitro and in vivo. Mol Cell. 1(5):639–648. doi:10.1016/S1097-2765(00)80064-7. 9660948

[jkae215-B66] Singer MA , Lindquist S. 1998b. Thermotolerance in *Saccharomyces cerevisiae*: the Yin and Yang of trehalose. Trends Biotechnol. 16(11):460–468. doi:10.1016/S0167-7799(98)01251-7. 9830154

[jkae215-B67] Sopko R , Huang D, Preston N, Chua G, Papp B, Kafadar K, Snyder M, Oliver SG, Cyert M, Hughes TR, et al 2006. Mapping pathways and phenotypes by systematic gene overexpression. Mol Cell. 21(3):319–330. doi:10.1016/j.molcel.2005.12.011. 16455487

[jkae215-B68] Stucka R , Blázquez MA. 1993. The fdp1 and cif1 mutations are caused by different single nucleotide changes in the yeast CIF1 gene. FEMS Microbiol Lett. 107(2–3):251–253. doi:10.1111/j.1574-6968.1993.tb06038.x. 8472906

[jkae215-B69] Su Y , Macías LG, Heras JM, Querol A, Guillamón JM. 2021. Phenotypic and genomic differences among *S. cerevisiae* strains in nitrogen requirements during wine fermentations. Food Microbiol. 96:103685. doi:10.1016/j.fm.2020.103685. 33494889

[jkae215-B70] Swinnen S , Klein M, Carrillo M, McInnes J, Nguyen HTT, Nevoigt E. 2013. Re-evaluation of glycerol utilization in *Saccharomyces cerevisiae*: characterization of an isolate that grows on glycerol without supporting supplements. Biotechnol Biofuels. 6(1):157. doi:10.1186/1754-6834-6-157. 24209984 PMC3835864

[jkae215-B71] Swulius MT , Jensen GJ. 2012. The helical mreb cytoskeleton in *Escherichia coli* MC1000/pLE7 is an artifact of the N-terminal yellow fluorescent protein tag. J Bacteriol. 194(23):6382–6386. doi:10.1128/JB.00505-12. 22904287 PMC3497537

[jkae215-B72] Tapia H , Young L, Fox D, Bertozzi CR, Koshland D. 2015. Increasing intracellular trehalose is sufficient to confer desiccation tolerance to *Saccharomyces cerevisiae*. Proc Natl Acad Sci U S A. 112(19):6122–6127. doi:10.1073/pnas.1506415112. 25918381 PMC4434740

[jkae215-B73] Thevelein JM . 1984. Regulation of trehalose mobilization in fungi. Microbiol Rev. 48(1):42–59. doi:10.1128/mr.48.1.42-59.1984. 6325857 PMC373002

[jkae215-B74] Van Dijck P , Colavizza D, Smet P, Thevelein JM. 1995. Differential importance of trehalose in stress resistance in fermenting and nonfermenting *Saccharomyces cerevisiae* cells. Appl Environ Microbiol. 61(1):109–115. doi:10.1128/aem.61.1.109-115.1995. 7887593 PMC167267

[jkae215-B75] Vanhalewyn M , Dumortier F, Debast G, Colombo S, Ma P, Winderickx J, Van Dijck P, Thevelein JM. 1999. A mutation in *Saccharomyces cerevisiae* adenylate cyclase, Cyr1(K1876M), specifically affects glucose- and acidification-induced cAMP signalling and not the basal cAMP level. Mol Microbiol. 33(2):363–376. doi:10.1046/j.1365-2958.1999.01479.x. 10411752

[jkae215-B76] Vind J , Sørensen MA, Rasmussen MD, Pedersen S. 1993. Synthesis of proteins in *Escherichia coli* is limited by the concentration of free ribosomes: expression from reporter genes does not always reflect functional mRNA levels. J Mol Biol. 231(3):678–688. doi:10.1006/jmbi.1993.1319. 7685825

[jkae215-B77] Walther T , Mtimet N, Alkim C, Vax A, Loret M-O, Ullah A, Gancedo C, Smits GJ, François JM. 2013. Metabolic phenotypes of *Saccharomyces cerevisiae* mutants with altered trehalose 6-phosphate dynamics. Biochem J. 454(2):227–237. doi:10.1042/BJ20130587. 23763276

[jkae215-B78] Warringer J , Zörgö E, Cubillos FA, Zia A, Gjuvsland A, Simpson JT, Forsmark A, Durbin R, Omholt SW, Louis EJ, et al 2011. Trait variation in yeast is defined by population history. PLoS Genet. 7(6):e1002111. doi:10.1371/journal.pgen.1002111. 21698134 PMC3116910

[jkae215-B79] Yi C , Wang F, Dong S, Li H. 2016. Changes of trehalose content and expression of relative genes during the bioethanol fermentation by *Saccharomyces cerevisiae*. Can J Microbiol. 62(10):827–835. doi:10.1139/cjm-2015-0832. 27510429

[jkae215-B80] Zähringer H , Thevelein JM, Nwaka S. 2000. Induction of neutral trehalase Nth1 by heat and osmotic stress is controlled by STRE elements and Msn2/Msn4 transcription factors: variations of PKA effect during stress and growth. Mol Microbiol. 35(2):397–406. doi:10.1046/j.1365-2958.2000.01706.x. 10652100

[jkae215-B81] Zhao G , Chen Y, Carey L, Futcher B. 2016. Cyclin-dependent kinase co-ordinates carbohydrate metabolism and cell cycle in *S. cerevisiae*. Mol Cell. 62(4):546–557. doi:10.1016/j.molcel.2016.04.026. 27203179 PMC4905568

