# Supplementary material for: Evaluating cellular roles and phenotypes associated with trehalose degradation genes in Saccharomyces cerevisiae
Source: G3 (Bethesda). 2024 Sep 9;14(11):jkae215. doi: 10.1093/g3journal/jkae215 (PMC11540316; doi:10.1093/g3journal/jkae215)
Supplement: jkae215_Supplementary_Data [file jkae215_supplementary_data.pdf]

Evaluating cellular roles and phenotypes associated with trehalose degradation genes in *Saccharomyces cerevisiae*

Anqi Chen[1], Sara E. Stadulis[1], Kayla deLeuze[1], Patrick A. Gibney[1]
[1]Department of Food Science, Cornell University, Ithaca, NY, 14853

SUPPLEMENTAL INFORMATION

Supplemental Table 1 Strains used in this study

Supplemental Table 2 Plasmids used in this study

Supplemental Table 3 Statistical analyses of the phenotypic differences between different strain wild types

Supplemental Table 4 Statistical analyses of the phenotypic differences between different strain *nth1Δ* mutants

Supplemental Table 5 Statistical analyses of the phenotypic differences between different strain *nth2Δ* mutants

Supplemental Table 6 Statistical analyses of the phenotypic differences between different strain *nth1Δnth2Δ* mutants

Supplemental Table 7 Statistical analyses of the phenotypic differences between different strain *ath1Δ* mutants

Supplemental Figure 1 PCR confirmation of heterozygous and homozygous mutant strains

Supplemental Figure 2 Trehalose degradation mutant cell sizes

Supplemental Figure 3 *nth1Δ, nth2Δ* and *nth1Δnth2Δ* mutant growth at 37°C

Supplemental Figure 4 *ath1Δ* mutant growth at 37°C

Supplemental Figure 5 Presence of *AGT1* in the genome does not affect growth on trehalose in *ath1Δ*

Supplemental Figure 6 Quiescent stationary phase trehalase mutants do not exhibit a lag phase defect

Supplemental Figure 7 Fates of dissected spores for strains in Figure 8D

Supplemental References

Supplemental Table 1. Strains used in this study.

Strain ID	Name in text	Genotype and/or description	Reference
DBY12007	S288C WT (diploid)	Prototrophic *HAP1*+ derivative of FY4	See below[a]
PGY423	S288C *nth1Δ/nth1Δ*	*nth1Δ*::kanMX/*nth1Δ*::kanMX	this study
PGY422	S288C *nth2Δ/nth2Δ*	*nth2Δ*::natAC/*nth2Δ*::natAC[b]	this study
DBY12640	S288C *nth1Δ/nth1Δ nth2Δ/nth2Δ*	*nth1Δ*::kanMX/*nth1Δ*::kanMX *nth2Δ*::natAC/*nth2Δ*::natAC	this study
PGY425	S288C *ath1Δ/ath1Δ*	*ath1Δ*::hphMX/*ath1Δ*::hphMX	this study
PGY439	S288C *nth1Δ/nth1Δ nth2Δ/nth2Δ ath1Δ/ath1Δ*	*nth1Δ*::kanMX/*nth1Δ*::kanMX *nth2Δ*::natAC/*nth2Δ*::natAC *ath1Δ*::hphMX/*ath1Δ*::hphMX	this study
PGY4	Simi White WT	Commercial wine strain provided by E&J Gallo Winery	Richter *et al.*, 2013
PGY354	Simi White *nth1Δ/nth1Δ*	*nth1Δ*::natAC/*nth1Δ*::natAC	this study
PGY380	Simi White *nth2Δ/nth2Δ*	*nth2Δ*::hphMX/*nth2Δ*::hphMX	this study
PGY404	Simi White *nth1Δ/nth1Δ nth2Δ/nth2Δ*	*nth1Δ*::natAC/*nth1Δ*::natAC *nth2Δ*::hphMX/*nth2Δ*::hphMX	this study
PGY388	Simi White *ath1Δ/ath1Δ*	*ath1Δ*::kanMX/*ath1Δ*::kanMX	this study
PGY441	Simi White *nth1Δ/nth1Δ nth2Δ/nth2Δ ath1Δ/ath1Δ*	*nth1Δ*::natAC/*nth1Δ*::natAC *nth2Δ*::hphMX/*nth2Δ*::hphMX *ath1Δ*::kanMX/*ath1Δ*::kanMX	this study
PGY34	YPS1000 WT	Natural isolate from New Jersey, United States; from oak exudate; provided by Joseph Schacherer lab	Sniegowski *et al.*, 2002
PGY345	YPS1000 *nth1Δ/nth1Δ*	*nth1Δ*::natAC/*nth1Δ*::natAC	this study
PGY374	YPS1000 *nth2Δ/nth2Δ*	*nth2Δ*::hphMX/*nth2Δ*::hphMX	this study
PGY407	YPS1000 *nth1Δ/nth1Δ nth2Δ/nth2Δ*	*nth1Δ*::natAC/*nth1Δ*::natAC *nth2Δ*::hphMX/*nth2Δ*::hphMX	this study
PGY390	YPS1000 *ath1Δ/ath1Δ*	*ath1Δ*::kanMX/*ath1Δ*::kanMX	this study
PGY525	YPS1000 *nth1Δ/nth1Δ nth2Δ/nth2Δ ath1Δ/ath1Δ*	*nth1Δ*::natAC/*nth1Δ*::natAC *nth2Δ*::hphMX/*nth2Δ*::hphMX *ath1Δ*::kanMX/*ath1Δ*::kanMX	this study
PGY7	CSM WT	Commercial wine strain provided by E&J Gallo Winery	Richter *et al.*, 2013
PGY351	CSM *nth1Δ/nth1Δ*	*nth1Δ*::natAC/*nth1Δ*::natAC	this study
PGY378	CSM *nth2Δ/nth2Δ*	*nth2Δ*::hphMX/*nth2Δ*::hphMX	this study
PGY400	CSM *nth1Δ/nth1Δ nth2Δ/nth2Δ*	*nth1Δ*::natAC/*nth1Δ*::natAC *nth2Δ*::hphMX/*nth2Δ*::hphMX	this study
PGY384	CSM *ath1Δ/ath1Δ*	*ath1Δ*::kanMX/*ath1Δ*::kanMX	this study
PGY442	CSM *nth1Δ/nth1Δ nth2Δ/nth2Δ ath1Δ/ath1Δ*	*nth1Δ*::natAC/*nth1Δ*::natAC *nth2Δ*::hphMX/*nth2Δ*::hphMX *ath1Δ*::kanMX/*ath1Δ*::kanMX	this study
PGY46	Bb32 WT	Natural isolate from Ravenswood Winery, California, United States; isolated by Robert Mortimer from Zinfandel in 1993; progenitor of RM11-1a; provided by Leonid Kruglyak lab	Mortimer *et al.*, 1994
PGY348	Bb32 *nth1Δ/nth1Δ*	*nth1Δ*::natAC/*nth1Δ*::natAC	this study
PGY376	Bb32 *nth2Δ/nth2Δ*	*nth2Δ*::hphMX/*nth2Δ*::hphMX	this study
PGY397	Bb32 *nth1Δ/nth1Δ nth2Δ/nth2Δ*	*nth1Δ*::natAC/*nth1Δ*::natAC *nth2Δ*::hphMX/*nth2Δ*::hphMX	this study
PGY382	Bb32 *ath1Δ/ath1Δ*	*ath1Δ*::kanMX/*ath1Δ*::kanMX	this study
PGY440	Bb32 *nth1Δ/nth1Δ nth2Δ/nth2Δ ath1Δ/ath1Δ*	*nth1Δ*::natAC/*nth1Δ*::natAC *nth2Δ*::hphMX/*nth2Δ*::hphMX *ath1Δ*::kanMX/*ath1Δ*::kanMX	this study
DBY12045	S288C *ura3Δ0*	MAT**a** *ura3Δ0*	Gibney *et al.*, 2020
PGY447	S288C *ura3Δ0 nth1Δ nth2Δ*	MAT**a** *ura3Δ0 nth1Δ*::kanMX *nth2Δ*::natAC	this study
DBY12000	S288C WT (haploid)	MAT**a** prototrophic *HAP1*+ derivative of FY4	See below[a]
PGY482	S288C *end3Δ*	MAT**a** *end3Δ*::kanMX	this study
DBY12313	S288C *ath1Δ*	MAT**a** *ath1Δ*::hphMX	Gibney *et al.*, 2015
DBY12431	S288C *ura3Δ0ath1Δ*	MAT**a** *ura3Δ0 ath1Δ*::hphMX	this study
PGY561	S288C *ATH1*-Green	MAT**a** *ath1::ATH1-linker-3XymNeonGreen-kanMX*	this study

a - All S288C-derived strains are *HAP1*-repaired, GAL$^+$, prototrophic derivatives of S288C. The details for constructing DBY12000 are found in *2007 MCB* Hickmann and Winston (3).
b-natAC refers to a version of the natMX dominant drug resistance marker cassette that contains a yeast codon-optimized nat^r gene. This cassette was generously provided by Amy Caudy.

Supplemental Table 2. Plasmids used in this study.

**Plasmid ID**	**Name in Text**	**Reference**
RB3269	pFA6a-kanMX (geneticin resistance)	Bähler *et al.*, 1998
RB3394	pAC372 (nourseothricin resistance)	Gibney *et al.*, 2015
RB3264	pAG32 (hygromycin B resistance)	Goldstein & McCusker, 1999
RB3620	p416GPD	Mumberg *et al.*, 1995; Sikorski & Hieter, 1989
RB3622	p426GPD	Mumberg *et al.*, 1995; Sikorski & Hieter, 1989
PGB106	p416GPD-*ATH1* (low-copy)	this study
PGB107	p426GPD-*ATH1* (high-copy)	this study
pGP564-YGPM4b20	Plasmid containing *AGT1/MAL11*	Jones *et al.*, 2008
pGP564-YGPM21p08	Plasmid containing *MAL31*	Jones *et al.*, 2008

All PGB plasmid inserts include a 5’ end SpeI site a 3’ end XhoI site for diagnostic digests.

SUPPLEMENTAL TABLE 3

Phenotypes	S288C WT	Simi White WT	YPS1000 WT	CSM WT	Bb32 WT
Cell size (fL)	41.7 ± 2.2^{a}	52.0 ± 1.4^{b}	51.1 ± 0.8^{bd}	47.8 ± 1.8^{c}	48.7 ± 0.3^{cd}
Trehalose level (μg glucose/OD unit)	7.6 ± 0.4^{a}	5.1 ± 0.6^{b}	3.7 ± 0.1^{c}	6.3 ± 0.1^{d}	7.7 ± 0.1^{a}
Glycogen level (μg glucose/OD unit)	1.7 ± 0.0^{a}	2.2 ± 0.1^{b}	1.2 ± 0.4^{c}	2.0 ± 0.1^{ab}	3.6 ± 0.1^{d}
Heat sensitivity (%)	94.2 ± 3.0^{a}	99.1 ± 0.6^{b}	81.8 ± 2.5^{c}	71.2 ± 1.1^{d}	98.1 ± 1.0^{b}
Sporulation efficiency (%)	71.2 ± 2.0^{a}	86.2 ± 2.6^{b}	96.3 ± 1.1^{c}	71.7 ± 1.3^{a}	85.9 ± 3.3^{b}

SUPPLEMENTAL TABLE 3. Statistical analysis of the phenotypic differences between wild type strains. Throughout the main text, results are primarily discussed as comparisons between mutant strains and their own isogenic wild type strains; assessments of statistically significant differences in the main text reflected those comparisons. As briefly discussed in the main text, it is also possible and potentially valuable to assess statistically significant differences between strains with similar gene deletions, or between the different wild type strains evaluated in this study. This table includes data from the wild type strains evaluated in this study. Values are identical to data presented in figures throughout the main text, and represent the means ± SD of 6 independent biological replicates. Cell growth and treatments are described in Materials and Methods, Results, and Figure Caption sections of the main text. Mean values in a row without a common superscript letter (a-d) represent statistically significant differences as analyzed by one-way ANOVA with *post hoc* Tukey's HSD test using a p-value threshold of less than 0.05 ($p < 0.05$).

SUPPLEMENTAL TABLE 4

Phenotypes	S288C *nth1*Δ	Simi White *nth1*Δ	YPS1000 *nth1*Δ	CSM *nth1*Δ	Bb32 *nth1*Δ
Cell size (fL)	39.4±1.9[a]	54.6±0.2[b]	50.5±0.2[c]	45.7±0.7[d]	48.8±1.3[c]
Trehalose level (μg glucose/OD unit)	10.0±0.2[a]	8.9±0.1[b]	8.4±0.3[c]	9.3±0.0[bd]	9.5±0.1[ad]
Glycogen level (μg glucose/OD unit)	2.2±0.1[a]	1.1±0.0[b]	1.8±0.1[a]	3.7±0.1[c]	4.8±0.5[d]
Heat sensitivity (%)	76.4±9.2[abc]	87.9±5.2[b]	70.9±2.9[cd]	64.4±4.5[c]	80.9±2.7[bd]
Sporulation efficiency (%)	70.8±2.3[a]	88.1±2.5[b]	97.2±0.2[c]	71.0±2.0[a]	88.3±1.0[b]

SUPPLEMENTAL TABLE 4. Statistical analysis of the phenotypic differences between *nth1Δ* strains. Throughout the main text, results are primarily discussed as comparisons between mutant strains and their own isogenic wild type strains; assessments of statistically significant differences in the main text reflected those comparisons. As briefly discussed in the main text, it is also possible and potentially valuable to assess statistically significant differences between strains with similar gene deletions, or between the different wild type strains evaluated in this study. This table includes data from the *nth1*Δ strains evaluated in this study. Values are identical to data presented in figures throughout the main text, and represent the means ± SD of 3 independent biological replicates. Cell growth and treatments are described in Materials and Methods, Results, and Figure Caption sections of the main text. Mean values in a row without a common superscript letter (a-d) represent statistically significant differences as analyzed by one-way ANOVA with *post hoc* Tukey's HSD test using a p-value threshold of less than 0.05 ($p < 0.05$).

SUPPLEMENTAL TABLE 5

Phenotypes	S288C *nth2*Δ	Simi White *nth2*Δ	YPS1000 *nth2*Δ	CSM *nth2*Δ	Bb32 *nth2*Δ
Cell size (fL)	38.8±0.2[a]	52.9±1.5[b]	50.1±1.9[be]	44.6±1.1[c]	48.8±1.0[de]
Trehalose level (μg glucose/OD unit)	9.6±0.4[a]	8.0±0.4[b]	5.6±0.3[c]	7.7±0.0[b]	9.1±0.1[a]
Glycogen level (μg glucose/OD unit)	1.6±0.0[a]	0.8±0.1[b]	1.4±0.0[c]	3.7±0.0[d]	4.8±0.0[e]
Heat sensitivity (%)	88.7±2.6[abc]	97.6±7.3[b]	78.4±1.3[ce]	67.4±4.4[de]	81.8±4.4[ac]
Sporulation efficiency (%)	70.1±2.0[a]	88.0±1.0[b]	97.3±0.6[c]	72.0±0.7[a]	87.7±1.0[b]

SUPPLEMENTAL TABLE 5. Statistical analysis of the phenotypic differences between *nth2Δ* strains. Throughout the main text, results are primarily discussed as comparisons between mutant strains and their own isogenic wild type strains; assessments of statistically significant differences in the main text reflected those comparisons. As briefly discussed in the main text, it is also possible and potentially valuable to assess statistically significant differences between strains with similar gene deletions, or between the different wild type strains evaluated in this study. This table includes data from the *nth2*Δ strains evaluated in this study. Values are identical to data presented in figures throughout the main text, and represent the means ± SD of 3 independent biological replicates. Cell growth and treatments are described in Materials and Methods, Results, and Figure Caption sections of the main text. Mean values in a row without a common superscript letter (a-d) represent statistically significant differences as analyzed by one-way ANOVA with *post hoc* Tukey's HSD test using a p-value threshold of less than 0.05 ($p < 0.05$).

SUPPLEMENTAL TABLE 6

Phenotypes	**S288C** ***nth1Δnth2Δ***	**Simi White** ***nth1Δnth2Δ***	**YPS1000** ***nth1Δnth2Δ***	**CSM** ***nth1Δnth2Δ***	**Bb32** ***nth1Δnth2Δ***
Cell size (fL)	39.4±0.4^{a}	53.7±0.3^{b}	47.9±1.0^{c}	44.6±0.1^{d}	50.5±2.0^{c}
Trehalose level (μg glucose/OD unit)	11.6±0.0^{a}	9.3±0.1^{b}	10.7±0.3^{ce}	10.9±0.2^{c}	10.2±0.2^{de}
Glycogen level (μg glucose/OD unit)	1.9±0.1^{a}	0.9±0.0^{b}	3.0±0.1^{c}	3.4±0.2^{d}	5.2±0.0^{e}
Heat sensitivity (%)	86.3±1.2^{a}	86.7±0.5^{a}	72.5±6.3^{b}	63.0±0.3^{c}	87.6±1.9^{a}
Sporulation efficiency (%)	68.8±2.0^{a}	86.9±1.6^{b}	97.3±0.9^{c}	72.0±1.3^{a}	87.0±0.3^{b}

SUPPLEMENTAL TABLE 6. Statistical analysis of the phenotypic differences between *nth1Δnth2Δ* strains. Throughout the main text, results are primarily discussed as comparisons between mutant strains and their own isogenic wild type strains; assessments of statistically significant differences in the main text reflected those comparisons. As briefly discussed in the main text, it is also possible and potentially valuable to assess statistically significant differences between strains with similar gene deletions, or between the different wild type strains evaluated in this study. This table includes data from the *nth1Δnth2Δ* strains evaluated in this study. Values are identical to data presented in figures throughout the main text, and represent the means ± SD of 3 independent biological replicates. Cell growth and treatments are described in Materials and Methods, Results, and Figure Caption sections of the main text. Mean values in a row without a common superscript letter (a-d) represent statistically significant differences as analyzed by one-way ANOVA with *post hoc* Tukey's HSD test using a p-value threshold of less than 0.05 ($p < 0.05$).

SUPPLEMENTAL TABLE 7

Phenotypes	S288C *ath1*Δ	Simi White *ath1*Δ	YPS1000 *ath1*Δ	CSM *ath1*Δ	Bb32 *ath1*Δ
Cell size (fL)	42.2±0.5[a]	42.7±0.4[a]	49.6±0.4[b]	50.8±0.8[b]	54.7±1.4[c]
Trehalose level (μg glucose/OD unit)	10.7±0.3[a]	10.7±0.3[a]	5.0±0.2[c]	6.5±0.2[b]	11.0±0.3[a]
Glycogen level (μg glucose/OD unit)	2.0±0.2[a]	1.5±0.0[b]	1.2±0.1[c]	0.7±0.1[d]	1.9±0.0[a]
Heat sensitivity (%)	94.2 ±1.3[a]	102.4±4.5[a]	80.3±5.6[b]	75.4±2.2[b]	101.2±2.0[a]
Sporulation efficiency (%)	69.9±2.5[a]	81.1±1.7[b]	93.1±2.5[c]	71.0±0.3[a]	81.6±3.9[b]

SUPPLEMENTAL TABLE 7. Statistical analysis of the phenotypic differences between *ath1Δ* strains. Throughout the main text, results are primarily discussed as comparisons between mutant strains and their own isogenic wild type strains; assessments of statistically significant differences in the main text reflected those comparisons. As briefly discussed in the main text, it is also possible and potentially valuable to assess statistically significant differences between strains with similar gene deletions, or between the different wild type strains evaluated in this study. This table includes data from the *ath1*Δ strains evaluated in this study. Values are identical to data presented in figures throughout the main text, and represent the means ± SD of 3 independent biological replicates. Cell growth and treatments are described in Materials and Methods, Results, and Figure Caption sections of the main text. Mean values in a row without a common superscript letter (a-d) represent statistically significant differences as analyzed by one-way ANOVA with *post hoc* Tukey's HSD test using a p-value threshold of less than 0.05 ($p < 0.05$).

SUPPLEMENTAL FIGURE 1

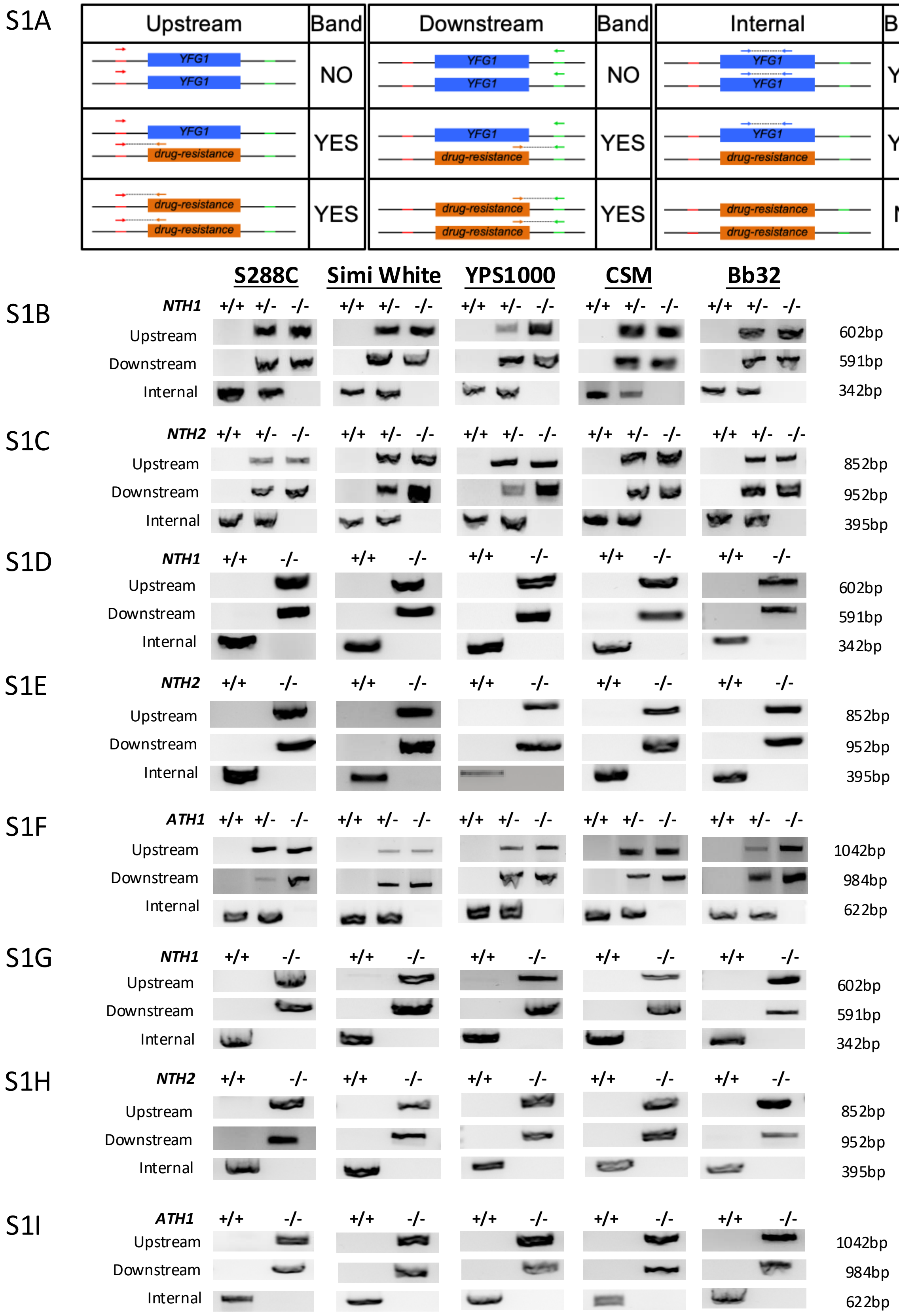

Supplemental Figure 1. PCR confirmation of heterozygous and homozygous mutant strains. A) PCR confirmation schematic graphic; **B)** Confirmation PCR image of *NTH1/nth1Δ* and *nth1Δ/nth1Δ* mutants; **C)** Confirmation PCR image of *NTH2/nth2Δ* and *nth2Δ/nth2Δ* mutants; **D, E)** Confirmation PCR image of *nth1Δ/nth1Δ nth2Δ/nth2Δ* mutants. **F)** Confirmation PCR image of *ATH1/ath1Δ* and *ath1Δ/ath1Δ* mutants; **G, H, I)** Confirmation PCR image of *nth1Δ/nth1Δ nth2Δ/nth2Δ ath1Δ/ath1Δ* mutants.

SUPPLEMENTAL FIGURE 2

S2A

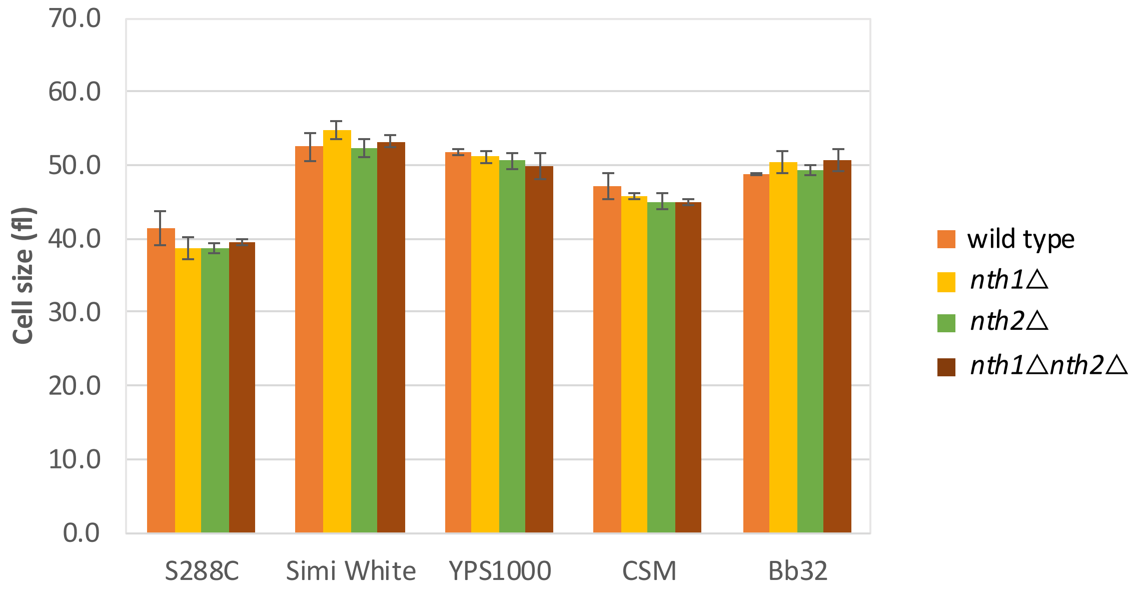

S2B

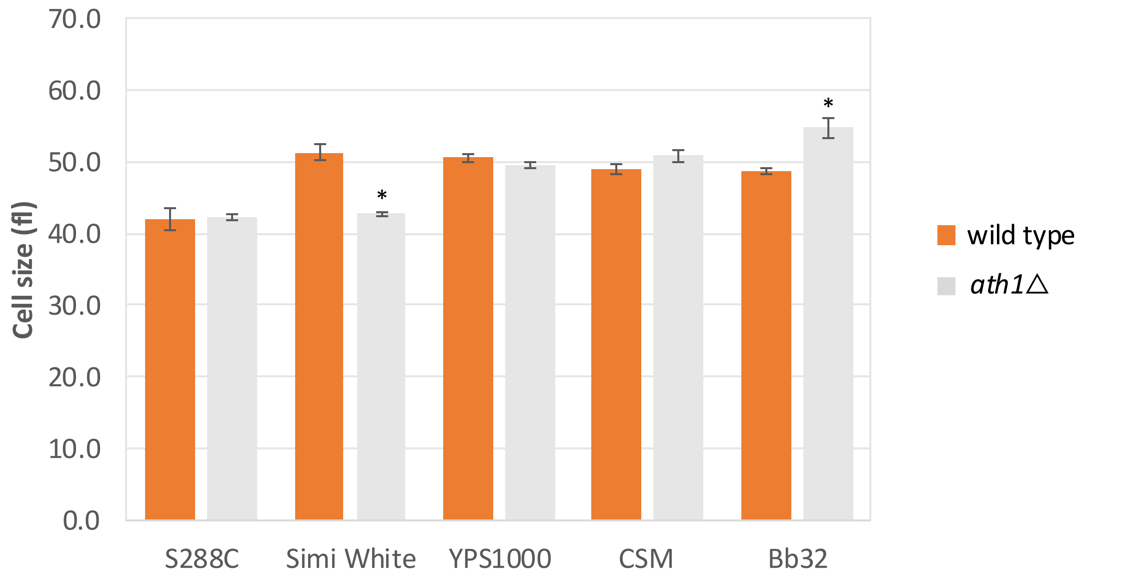

Supplemental Figure 2. Trehalose degradation mutant cell sizes. Cell size was measured using a Coulter counter as described in Materials and Methods. **A)** *nth1Δ, nth2Δ,* and *nth1Δnth2Δ* mutants; **B)** *ath1Δ* mutants. Three biological replicates were performed for each measurement and the values are presented as the mean ± standard deviation. Asterisks represent statistical difference ($p<0.05$) between the mutants and their isogenic wild types.

SUPPLEMENTAL FIGURE 3

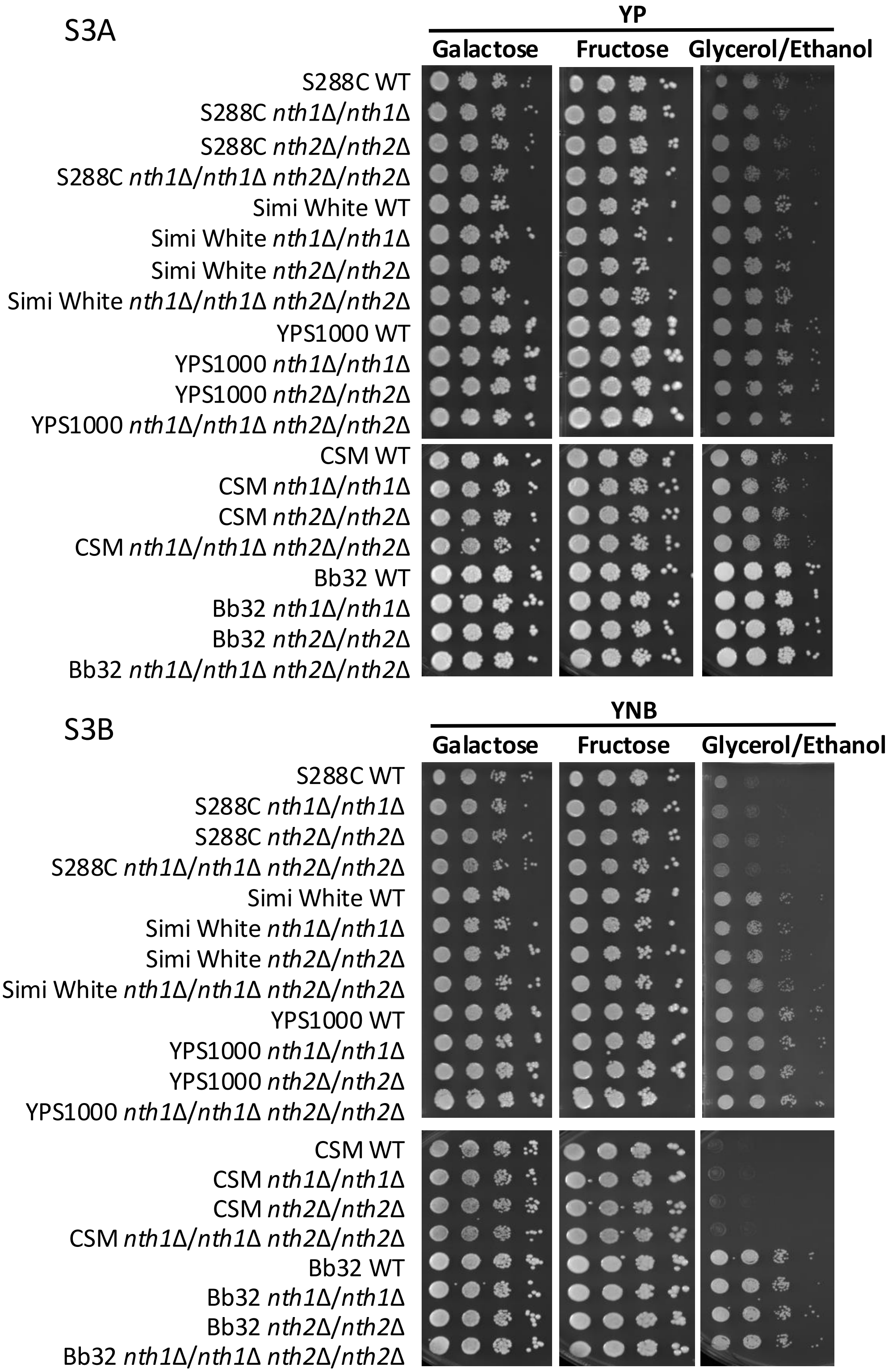

Supplemental Figure 3. *nth1Δ, nth2Δ,* and *nth1Δnth2Δ* mutant growth at 37°C. The indicated strains were grown overnight in YNB + 2% glucose liquid before 10-fold serial dilutions were prepared and spotted onto **A)** rich media or **B)** minimal media containing the indicated carbon sources. Galactose, fructose, and ethanol were present at 2%, glycerol was present at 3%. The initial dilution had an OD_{600} of 1.0. Plates were incubated at 37°C for 2 days on rich media and 3 days on minimal media. At least three biological replicates were performed, and a representative example is shown.

SUPPLEMENTAL FIGURE 4

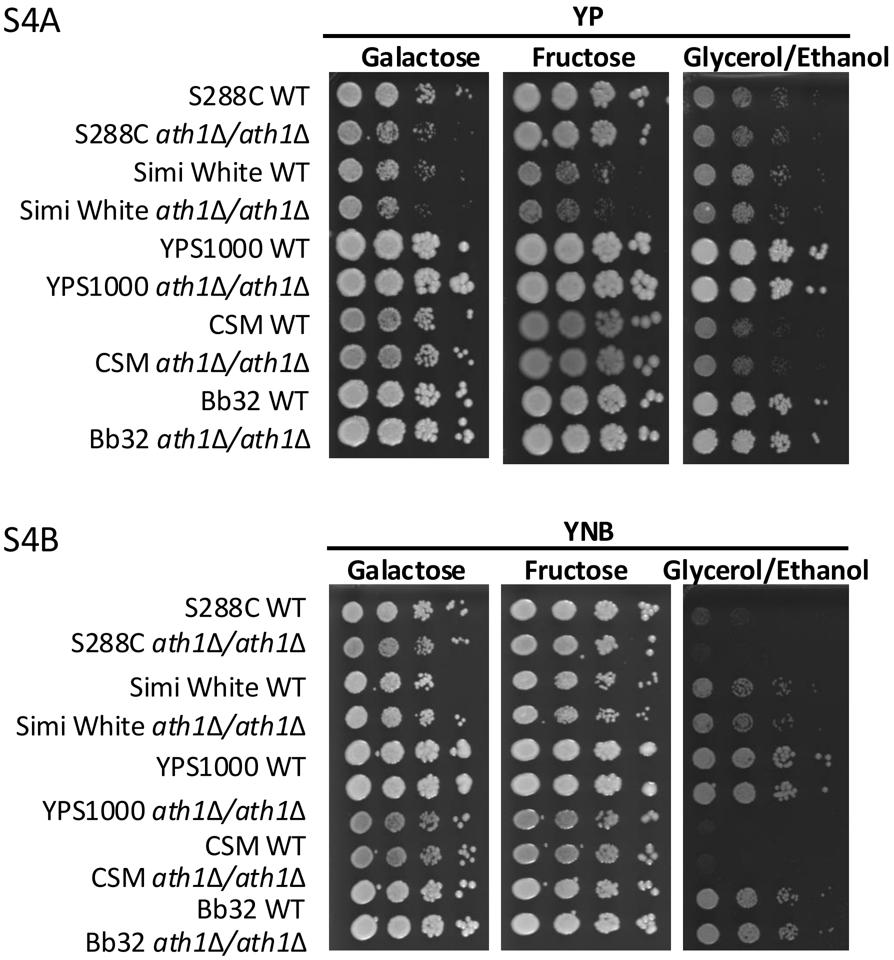

Supplemental figure 4. ***ath1Δ*** **mutant growth at 37°C.** The indicated strains were grown overnight in YNB + 2% glucose liquid before 10-fold serial dilutions were prepared and spotted onto **A)** rich media or **B)** minimal media containing the indicated carbon sources. Galactose, fructose, and ethanol were present at 2%, glycerol was present at 3%. The initial dilution had an OD_{600} of 1.0. Plates were incubated at 37°C for 2 days on rich media and 3 days on minimal media. At least three biological replicates were performed, and a representative example is shown.

SUPPLEMENTAL FIGURE 5

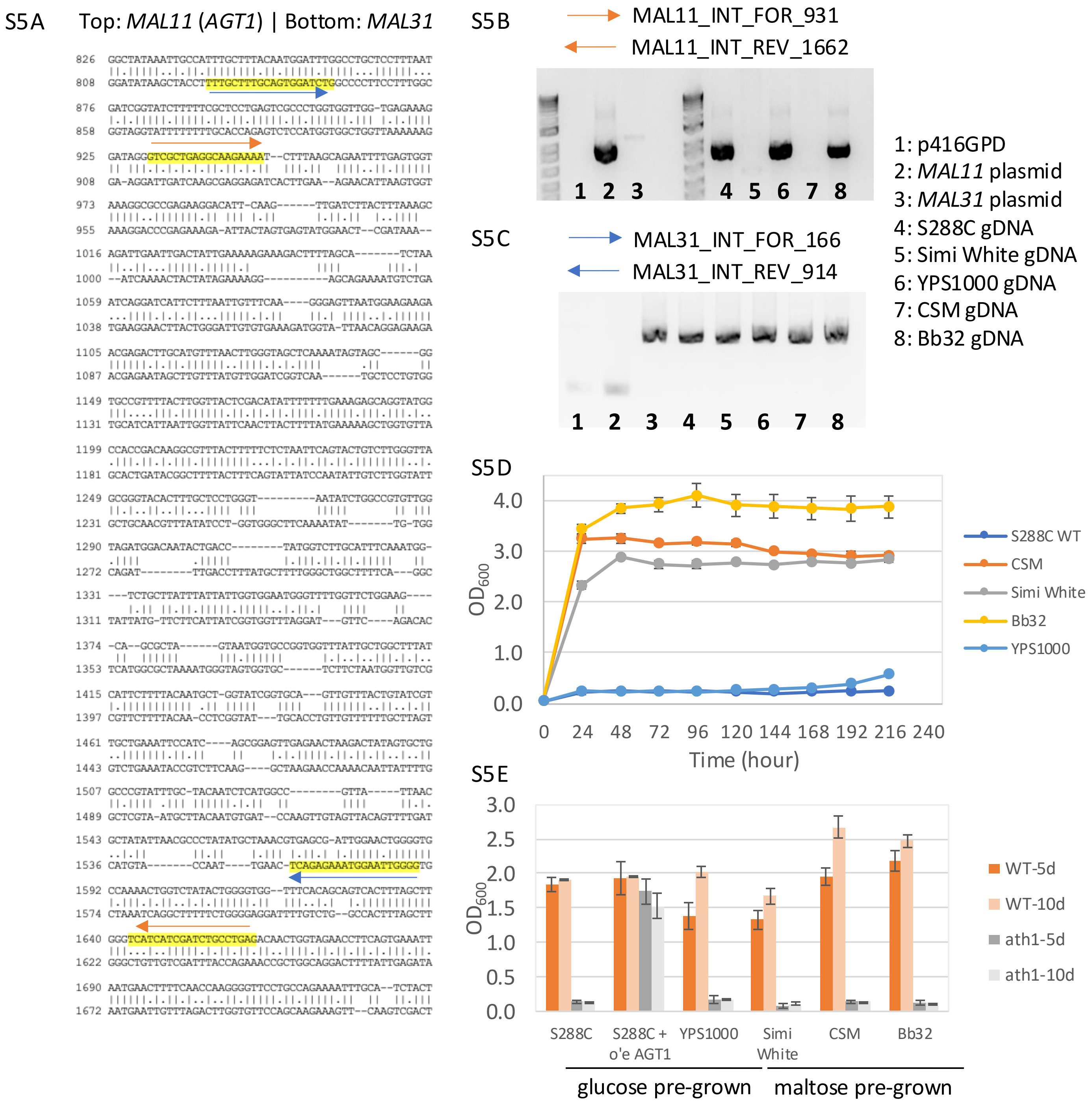

Supplemental Figure 5. Presence of *AGT1* in the genome does not affect growth on trehalose in *ath1Δ*. A) Gene sequence alignment of *MAL11*(*AGT1*) and *MAL31.* Arrows and highlighted bases indicate where confirmation primers bind; **B)** PCR confirmation of *MAL11*(*AGT1*); **C)** PCR confirmation of *MAL31*; **D)** Growth of five yeast strains in minimal maltose media. Cells were first inoculated into YNB + 2% glucose, incubated overnight, washed once with water and subcultured to OD_{600} of 0.05 into YNB + 2% maltose. OD_{600} was recorded every day for 10 days; **E)** S288C, S288C with overexpressed *AGT1* and YPS1000 strains were pre-grown in YNB + 2% glucose before diluted to OD_{600} of 0.05 and transferred into YNB + 1% trehalose; Simi White, CSM and Bb32 strains were pre-grown in YNB + 2% maltose before diluted to OD_{600} of 0.05 and transferred into YNB + 1% trehalose; OD600 was measured at 5 and 10 days. Three biological replicates were performed and the resulting values are presented as the mean ± standard deviation.

SUPPLEMENTAL FIGURE 6

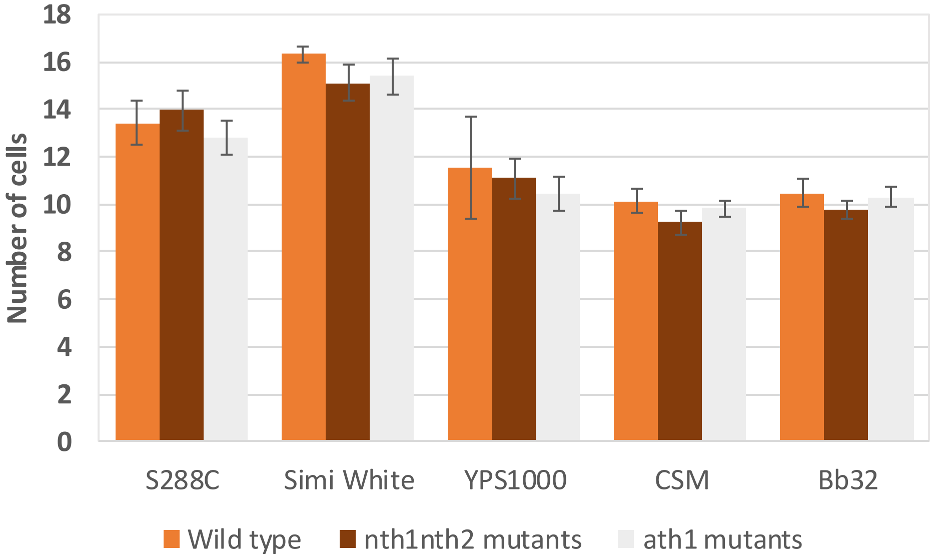

Supplemental Figure 6. Quiescent stationary phase trehalase mutants do not exhibit a lag phase defect. Quiescent, stationary phase wild type, *nth1*Δ*nth*2Δ, and *ath1*Δ cells were prepared by growth/incubation in YPD for 7 days at 30°C (Gasch and Werner-Washburne, 2002). Sixteen individual cells from each strain were placed at a unique location on a YPD plate using the micromanipulator needle on a tetrad dissection microscope. After 6 hours of incubation at 30°C, the number of resulting cells at each unique location was counted. The values are presented as the mean±standard deviation from 16 initial cells from each indicated strain.

SUPPLEMENTAL FIGURE 7

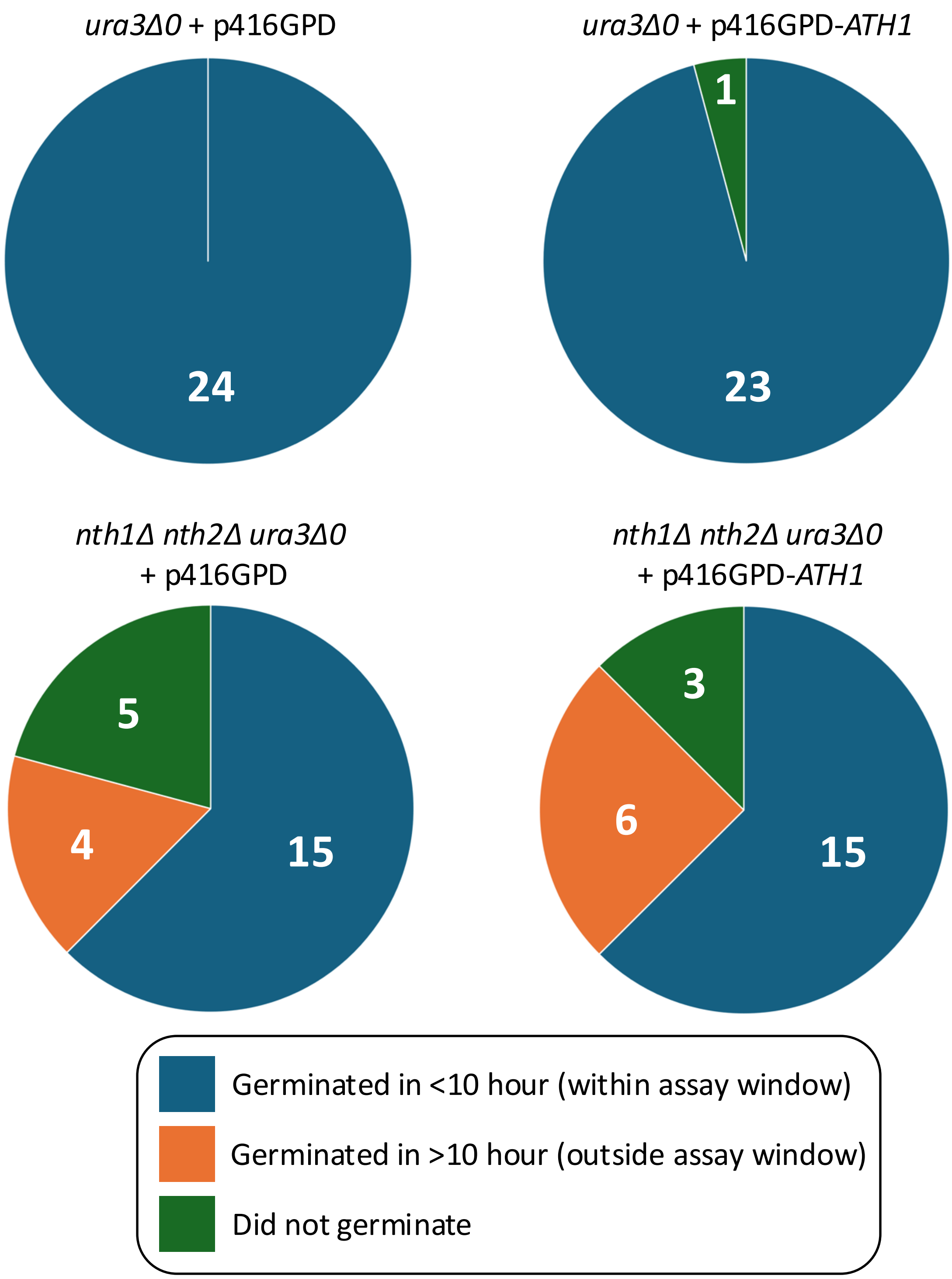

Supplemental Figure 7. Fates of dissected spores for strains in Figure 8D. Listed strains were evaluated as described in Materials and Methods for germination by scoring the amount of time required for initial bud emergence from a germinated spore. For each strain, n=24 spores: 2 tetrads (8 spores) each from 3 independent, replicate cultures. Data shown in Figure 8D represents bud emergence time for each strain within the 10-hour assay window. For spores scored as "did not germinate", plates were evaluated for germination through 48-hours of incubation at 30C.

Supplemental References

1. Mumberg D, Müller R, Funk M. Yeast vectors for the controlled expression of heterologous proteins in different genetic backgrounds. *Gene*. 1995;156(1):119-122.
2. Sikorski RS, Hieter P. A system of shuttle vectors and yeast host strains designed for efficient manipulation of DNA in Saccharomyces cerevisiae. *Genetics*. 1989;122(1):19-27.
3. Jones GM, Stalker J, Humphray S, et al. A systematic library for comprehensive overexpression screens in Saccharomyces cerevisiae. *Nat Methods*. 2008;5(3):239-241.
4. Gibney PA, Chen A, Schieler A, et al. A tps1Δ persister-like state in Saccharomyces cerevisiae is regulated by MKT1. PLoS One. 2020;15(5):e0233779.
5. Gibney PA, Schieler A, Chen JC, Rabinowitz JD, Botstein D. Characterizing the in vivo role of trehalose in Saccharomyces cerevisiae using the AGT1 transporter. *Proc Natl Acad Sci U S A*. 2015;112(19):6116-6121.
6. Richter CL, Dunn B, Sherlock G, Pugh T. Comparative metabolic footprinting of a large number of commercial wine yeast strains in Chardonnay fermentations. *FEMS Yeast Res*. 2013;13(4):394-410.
7. Sniegowski PD, Dombrowski PG, Fingerman E. Saccharomyces cerevisiae and Saccharomyces paradoxus coexist in a natural woodland site in North America and display different levels of reproductive isolation from European conspecifics. *FEMS Yeast Res*. 2002;1(4):299-306.
8. Mortimer RK, Romano P, Suzzi G, Polsinelli M. Genome renewal: a new phenomenon revealed from a genetic study of 43 strains of Saccharomyces cerevisiae derived from natural fermentation of grape musts. *Yeast*. 1994;10(12):1543-1552.
9. Bähler J, Wu JQ, Longtine MS, et al. Heterologous modules for efficient and versatile PCR-based gene targeting in Schizosaccharomyces pombe. *Yeast*. 1998;14(10):943-951.
10. Goldstein AL, McCusker JH. Three new dominant drug resistance cassettes for gene disruption in Saccharomyces cerevisiae. *Yeast*. 1999;15(14):1541-1553.
11. Gasch AP, Werner-Washburne M. The genomics of yeast responses to environmental stress and starvation. *Funct Integr Genomics*. 2002;2(4-5):181-192.
12. Hickman MJ, Winston F. Heme levels switch the function of Hap1 of Saccharomyces cerevisiae between transcriptional activator and transcriptional repressor. Mol Cell Biol. 2007;27: 7414–24.
